# The Differential Expression of Immune Genes between Water Buffalo and Yellow Cattle Determines Species-Specific Susceptibility to *Schistosoma japonicum* Infection

**DOI:** 10.1371/journal.pone.0130344

**Published:** 2015-06-30

**Authors:** Jianmei Yang, Zhiqiang Fu, Yang Hong, Haiwei Wu, Yamei Jin, Chuangang Zhu, Hao Li, Ke Lu, Yaojun Shi, Chunxiu Yuan, Guofeng Cheng, Xingang Feng, Jinming Liu, Jiaojiao Lin

**Affiliations:** 1 Shanghai Veterinary Research Institute, Chinese Academy of Agricultural Sciences, Key Laboratory of Animal Parasitology, Ministry of Agriculture of China, Shanghai, People’s Republic of China; 2 Department of Pathology and Laboratory Medicine, Alpert Medical School, Brown University, Providence, Rhode Island, United States of America; 3 Jiangsu Co-innovation Center for Prevention and Control of Important Animal Infectious Diseases and Zoonoses, Yangzhou, People’s Republic of China; Institut national de la santé et de la recherche médicale - Institut Cochin, FRANCE

## Abstract

Water buffalo are less susceptible to *Schistosoma japonicum* infection than yellow cattle. The factors that affect such differences in susceptibility remain unknown. A *Bos taurus* genome-wide gene chip was used to analyze gene expression profiles in the peripheral blood of water buffalo and yellow cattle pre- and post-infection with *S*. *japonicum*. This study showed that most of the identified differentially expressed genes(DEGs) between water buffalo and yellow cattle pre- and post-infection were involved in immune-related processes, and the expression level of immune genes was lower in water buffalo. The unique DEGs (390) in yellow cattle were mainly associated with inflammation pathways, while the unique DEGs (2,114) in water buffalo were mainly associated with immune-related factors. The 83 common DEGs may be the essential response genes during *S*. *japonicum* infection, the highest two gene ontology (GO) functions were associated with the regulation of fibrinolysis. The pathway enrichment analysis showed that the DEGs constituted similar immune-related pathways pre- and post-infection between the two hosts. This first analysis of the transcriptional profiles of natural hosts has enabled us to gain new insights into the mechanisms that govern their susceptibility or resistance to *S*. *japonicum* infections.

## Introduction

Schistosomiasis remains an important global public health problem that affects 200 million people in 76 countries. Schistosomiasis control in China has been remarkably successful, with the number of cases being reduced from 11,000,000 to 286,836 by the end of 2011 [1]. Endemic areas of uncontrolled schistosomiasis in China are mostly distributed in the marsh, lake, and mountainous regions [2,3]. Studies have shown that bovine infection is one of the main threats for human infection [4,5]. Water buffalo(*Bubalus bubalis*) and yellow cattle(*Bos indicus*) are considered to be the main transmission sources for schistosomiasis in China [6,7].

*Schistosoma japonicum* has a wide range of host species, and at least 46 species of mammals, other than humans, are known to be naturally infected by *S*. *japonicum*. The degree of susceptibility to *S*.*japonicum* has been previously demonstrated to vary among several host species [8]. In addition, host self-curing and parasite clearance have been observed in water buffalo and pigs after a certain period of infection [9,10]. Parasites that survive in such less-susceptible hosts also showed substantial changes in morphology, being shorter in length, having poorly developed gonads, and demonstrating a lower rate of worm pairing and spawning by female worms [11,12].

As a consequence of extensive host–parasite co-evolution, parasites exhibit a complex relationship with their hosts. Although the susceptibility/resistance mechanism for host infection with parasites remains unknown, immunological factors have proved to be intimately linked with parasite development [13,14]. Recently, there has been considerable interest in defining genetic and immunological markers that could be important for disease resistance [15,16].

In our lab, we have compared the gene expression profiles of schistosomes from two natural hosts, water buffalo and yellow cattle [17]. In this study, we used a bovine whole genome microarray to analyze the host molecular mechanism against schistosome infection. We report the first analysis of the transcriptional profiles of genes in peripheral blood from water buffalo and yellow cattle pre- and 7 weeks post-infection with *S*.*japonicum*. Understanding the molecular mechanisms affecting and regulating the development and survival of schistosomes will be essential for improving our knowledge of the host–pathogen relationship, revealing different susceptibilities within the natural host environment, and providing new ideas for the prevention of this disease.

## Materials and Methods

### Ethics Statement

All work was conducted in accordance with the guidelines of the Association for Assessment and Accreditation of Laboratory Animal Care International (AAALAC). The animal study protocol was approved by the Animal Care and Use Committee of the Shanghai Veterinary Research Institute, Chinese Academy of Agricultural Sciences (CAAS), People’s Republic of China.

### Collection of blood samples and RNA preparation

Water buffalo and yellow cattle (three per group, 15–18-month-old males) were infected with cecariae of *S*.*japonicum* according to the method described in previous reports [17, 18]. Before infection and 7 weeks post-infection, peripheral blood samples were collected from each animal using ordinary vacuum tubes containing a sterile anticoagulant EDTA dipotassium salt. RNA extraction of blood samples was completed within 24h. Total RNA was extracted and purified using the RNeasy Micro Kit and RNase-Free DNase Set (Qiagen, Germany) according to the manufacturer’s instructions. RNA samples were amplified and labeled using the Low Input Quick Amp Labeling Kit (Agilent Technologies, USA) as follows. A 200-ng aliquot of total RNA from each sample was converted into complementary RNA, labeled with the fluorophore cyanine 3-CTP (CY3c) and hybridized according to the manufacturer’s instructions. Samples were examined at A260 and A550 using a ND-1000 spectrophotometer (Thermo Scientific, USA) to determine the yield, concentration, amplification efficiency, and abundance of CY3c. The labeled complementary RNA (cRNA) was further purified using the RNeasy MiniKit (Qiagen).

### Microarray composition

A whole genome microarray (Bovine V2, Agilent Technologies) was used to analyze gene expression profiles in peripheral blood from water buffalo and yellow cattle pre- and 7 weeks post-infection with *S*.*japonicum*. The microarray design was based on the sequence data of Btau 4.0 (Refseq Release 34, Mar 2009[19]; TIGR Release12, Sep 2006; UniGene Build 93, Sep 2008), 60-merSurePrint technology, and included 43,803 contiguous sequences (contigs), printed in a 4×44 k feature format. Full details of this bovine microarray design have been deposited in the Gene Expression Omnibus (GEO) public database with the platform accession number GPL11648.

### Microarray hybridization

For the samples from each host, three independent biological replicates were designed for microarray hybridization. The samples from yellow cattle were named Group 1 (g1) and Group 2 (g2) for pre- and 7 weeks post-infection, respectively; the samples from water buffalo were named Group 3 (g3) and Group 4 (g4). The microarray hybridization experiments were fulfilled by Shanghai Biotechnology Corporation (China). All procedures were operated according to the standard processes and supporting kits provided for Agilent microarray hybridization.

### Feature extraction and data analysis

Microarrays were scanned using an Agilent Microarray Scanner (G2565CA) and processed with Feature Extraction Software 10.7 (Agilent Technologies) to produce standardized data for statistical analysis. Feature-extracted data were analyzed using GENESPRING (version 11.0; Agilent Technologies/Silicon Genetics, USA). Data sets were further analyzed based on one-color experiments published previously [20]. The gProcessed Signal values were determined in GENESPRING using Agilent’s Feature Extraction software. The gProcessed Signal represents the signal after localized background subtraction and includes corrections for surface trends. Features were deemed ‘Absent’ when the processed signal intensity was less than twice the value of the processed signal error value; ‘Marginal’ when the measured intensity was at a saturated value or if there was a substantial amount of variation in the signal intensity within the pixels of a particular feature. Features that were neither Absent nor Marginal were deemed ‘Present’. Data points were included only if they were Present or Present-Absent, and probes or contigs were retained if all data points were Present or Present-Absent.

The statistical analysis between two groups was performed using the Student’s test. Raw intensity data were analyzed using the R statistical language software (www.r-project.org). The *q-*value estimation for false discovery rate (FDR) control was applied to analyze the data for enrichment analysis of Go function and KEGG pathway (See S3, S4, S6, S8 and S9 Tables) [21, 22]. Heatmap and principal component analysis (PCA) were plotted using Java Treeview software (Stanford University, USA) and a multidimensional scaling algorithm [23].

### Gene ontology and pathway pattern analysis

Those genes considered as differentially expressed genes (DEGs), i.e., those having at least two-fold changes in expression between two groups, were analyzed further. The analysis was performed online at http://www.ebioservice.com/(supported by Shanghai Biochip Corporation, China), including screening DEGs, hierarchical clustering analysis, gene ontology (GO), and pathway pattern analysis. The analysis of GO terms associated with the DEGs in peripheral blood from the two host groups was performed using the combined graphs function of the BLAST software [24]. GO correlations with relative gene expression values were made using Ermine J software [25]. The Kyoto Encyclopedia of Genes and Genomes (KEGG) pathway of DEGs was analyzed using the maps at http://www.genome.jp/kegg.

### Real-time PCR

A subset of genes predicted to be DE in the microarray analysis was selected for validation using real-time RT-PCR. Gene-specific primers were designed using PRIMER3 (http://frodo.wi.mit.edu/primer3/input.htm). Total amplified RNA from peripheral blood samples from each animal in every group was used for reverse transcription (RT) in a final volume of 20 μL using the PrimerScript RT Kit with gDNA Eraser (Cat# DRR047, Takara, Japan). Products were amplified using the SYBR Premix Ex Taq (Cat#DRR041A, Takara) in an ABI 7500 Real-time System (Applied Biosystems, USA) with the following profile: 50°C for 2 min, 95°C for 30 s; 40 cycles of 95°C for 5 s and 60°C for 34 s; 95°C for 15 s, and 60°C for 1 min. Each reaction was performed using 20 μL of cDNA from the RT reaction in a final volume of 50 μL. Expression levels of *Bos taurus* beta-actin (ACTB, GenBank Accession NM_173979) were used as an endogenous control within each sample. Relative levels of gene expression were calculated using the 2^-ΔΔCT^ method [26]. Each sample was analyzed for primer dimers, contamination, mispriming and specificity by advanced inspection of their melt curves and gel images. All the samples were done by three repeats, the analysis tool for qPCR result was ABI 7500 PCR system software (SDS V1.4). The correlation between real-time PCR and microarray data was performed by SPSS16.0 using Spearman’s Rho measure of correlation [27].

## Results

### Global gene expression profiles of the microarray

**Fig 1:**
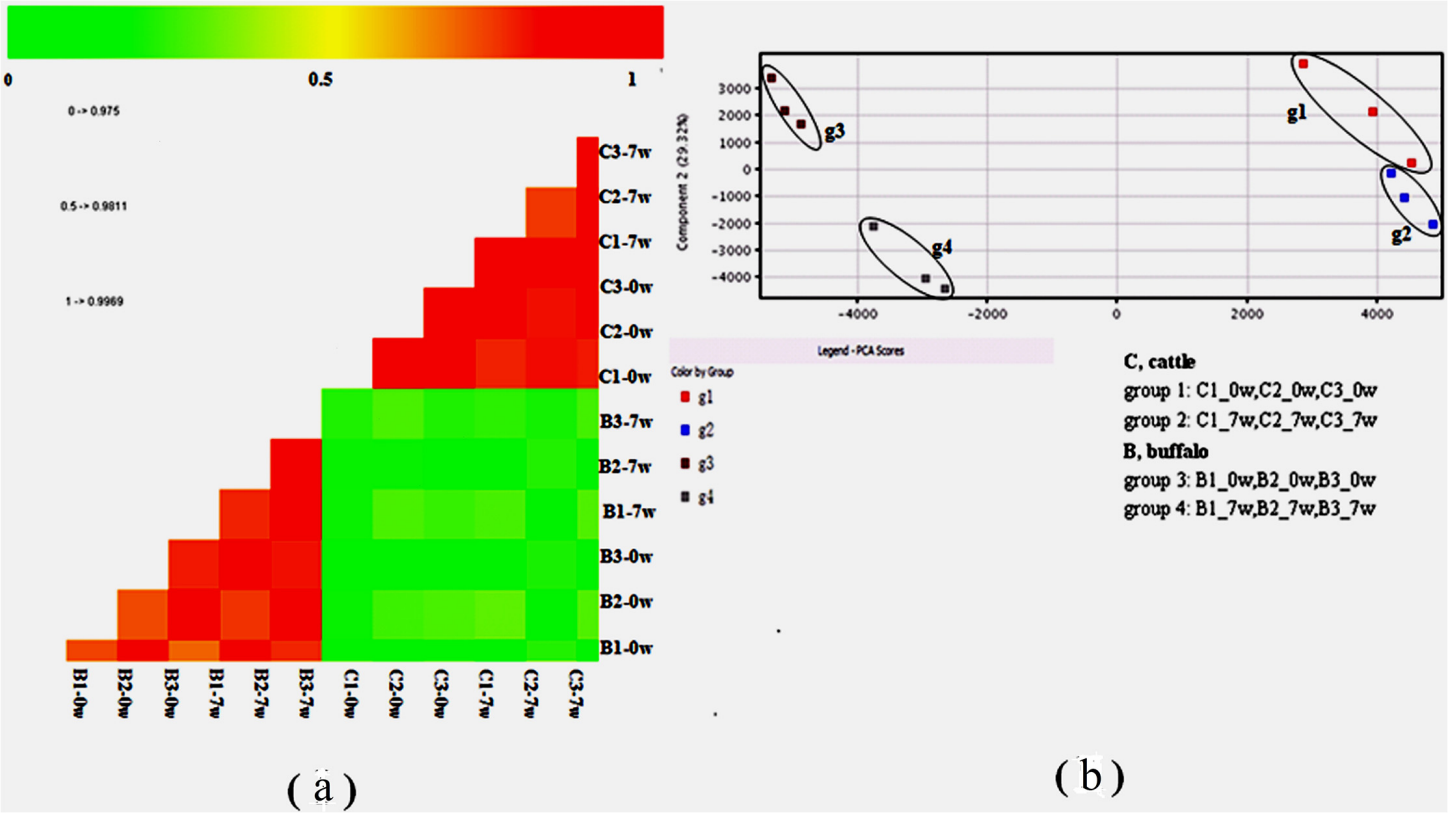
Global microarray data analysis. (a) Correlation analysis for all samples; (b) Principal component analysis (PCA) of the transcript profiles from g1, g2, g3 and g4. g1 and g2 represent the samples from pre-infection and post-infection, respectively, in yellow cattle (C, cattle);g3 and g4 represent the samples from pre-infection and post-infection, respectively, in water buffalo (B, buffalo).

**Fig 2:**
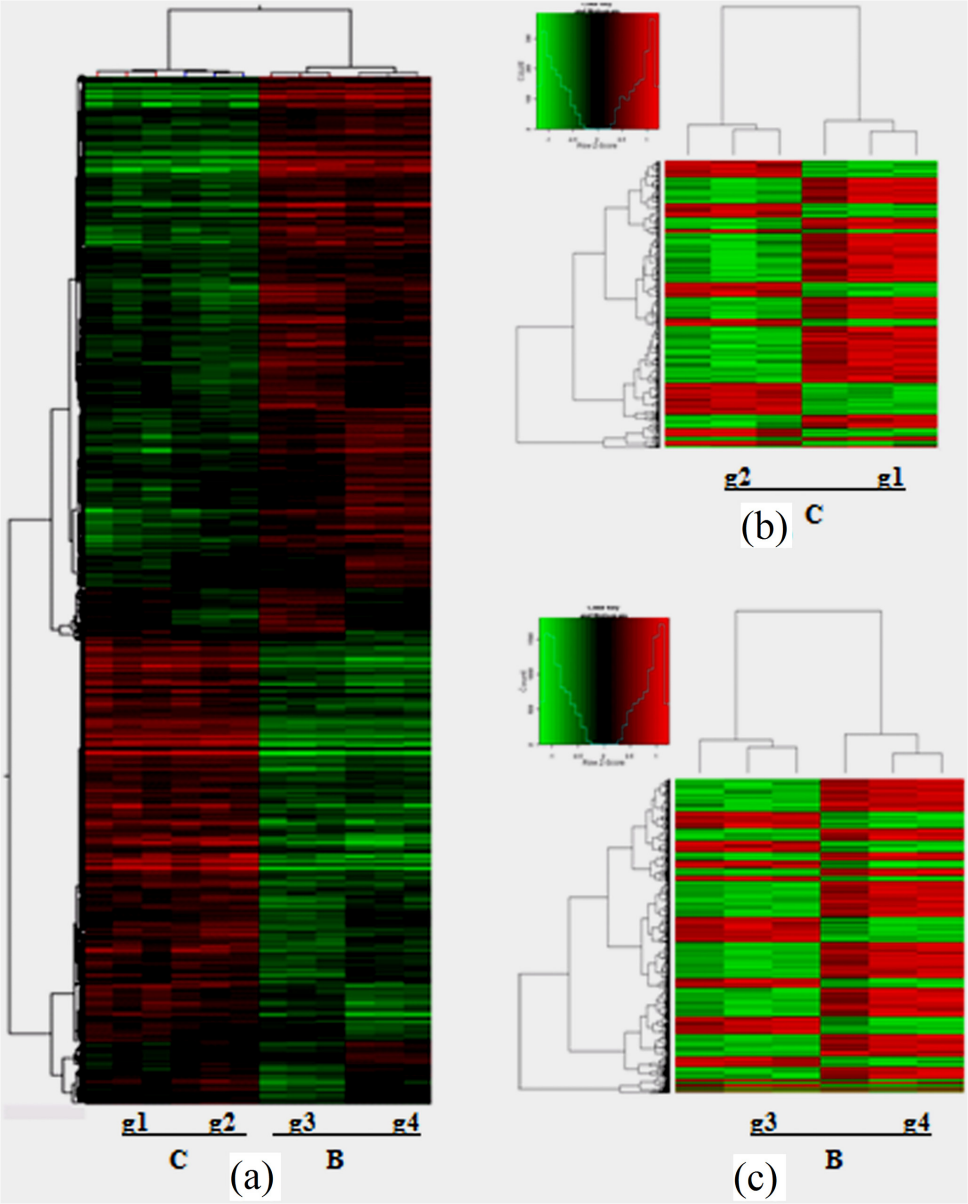
Cluster analysis for DEGs. (a) Cluster analysis for g1, g2, g3 and g4 (p<0.01, top 5,000); (b) Cluster analysis for g1 and g2 (p<0.01); (c) Cluster analysis for g3 and g4 (p<0.01).

Three biological replicates of blood samples from each host were evaluated and the correlation among a total of 12 samples was 0.97–0.99 (Fig 1A). The microarray data were submitted to the Gene Expression Omnibus public database, under the GEO series accession number: GSE34021 (http://www.ncbi.nlm.nih.gov/geo). To evaluate the overall data structure, we plotted the first two principal components of the PCA to capture the overall variance of all samples in two dimensions. This analysis clearly separated the data into four subgroups, which clustered the biological replicates together and separated the samples by the host and the infection time, i.e., yellow cattle (g1 and g2), water buffalo(g3 and g4) (Fig 1B). Two main clusters separated the genes of the two natural hosts, and the gene expression patterns of samples pre-infection and post-infection in each host group were clustered (Fig 2A). In the yellow cattle groups and water buffalo groups, significantly DEGs for hierarchical clustering were drawn by heatmap using Genespring software separately (fold change >2, p<0.01) (Fig 2B&2C).

### DEGs between water buffalo and yellow cattle pre-infection

Between water buffalo and yellow cattle, there were 5,740 DEGs at week zero. Compared with the samples from yellow cattle, there were 2,594 up-regulated and 3,146 down-regulated DEGs in water buffalo at week zero. Before infection, compared with yellow cattle, the up-regulated genes in the water buffalo group were mainly associated with tight junctions and the TGF-beta signaling pathway, while the down-regulated genes in the water buffalo group were mainly associated with the p53 signaling pathway, B cell receptor signaling pathway, T cell receptor signaling pathway, hematopoietic cell lineage, Toll-like receptor signaling pathway, and natural killer cell-mediated cytotoxicity (Table 1). In detail, the up-regulated genes in the water buffalo group included: IL-22R, IL-24, IL-17B, protein kinase C, growth differentiation factor 7, protein phosphatase 2A (S1 Table), while the down-regulated genes in the water buffalo group included: the MHC-I molecule, interferon family, IL-2R, IL-4R, CD80, CD46, TNF family (TNFSF5, TNFSF8), vascular endothelial growth factor C (S2 Table). In the cytokine–cytokine receptor interaction pathway, the DEGs in the interferon family, hematopoietins, chemokines, and TNF family were under-expressed in water buffalo, while the IL-10 family was over-expressed compared with yellow cattle before infection (Table 2). In total, before infection, the DEGs were mainly associated with immune-related pathways, and these differences are likely responsible for the resistance of water buffalo to schistosome infections.

**Table 1:** Functional pathways of DEGs in water buffalo compared with yellow cattle, pre-infection^a^.

Pathway	Genes included in pathway
**Pathway of up-regulated genes**	
Tight junction	ACTIN1,CLDN10,HCLS1,MPDZ,MRK,MYH3,MYH4,PPP2R1A,PPKCB,PRKC1
TGF-beta signaling pathway	DCN,GDF7,LEFTY2,PPP2R1A
**Pathway of down-regulated genes**	
p53 signaling pathway	CCNB1,CCNE2,CDK1,CDK4,SESN3
B cell receptor signaling pathway	IFITM1,IKBKG,JUN,LYN
T cell receptor signaling pathway	CDK4,IKBKG,JUN,MAPK13
Hematopoietic cell lineage	IL12RA,IL4R,ITGA4
Toll-like receptor signaling pathway	CD40,CD80,IFNAR1,IKBKG,JUN,LBP,MAPK13
Natural killer cell mediated cytotoxicity	IFNGR1,IFNGR2,JSP.1,GZMB,IFNAR1,SH2D1A

^a^ More details are provided in S1&S2 Tables.

**Table 2:** Some DEGs involved in cytokine–cytokine receptor interaction pathways in water buffalo compared with yellow cattle, pre-infection.

Gene ID	Symbol	Probe ID	p value	g3_vs_g1fold change	Gene name
**Interferon family**					
508619	IFNGR1	A_73_115260	3.0E-4	1.44E-3	interferon gamma receptor 1
514889	IFNGR2	A_73_107789	0.0	5.39E-3	interferon gamma receptor 2
282257	IFNAR1	A_73_116078	0.035	0.24	interferon alpha receptor 1
**Hematopoietins**					
281861	IL2RA	A_73_109472	0.0042	0.07	interleukin 2 receptor, alpha
404154	IL4R	A_73_112133	0.0014	0.37	interleukin 4 receptor
**Chemokines**					
281735	CXCL5	A_73_106079	0.024	0.22	chemokine (C-X-C motif) ligand 5
616732	CCL16	A_73_105123	7.0E-4	0.08	chemokine (C-C motif) ligand 16
408018	CCR3	A_73_103766	0.0090	0.16	chemokine (C-C motif) receptor 3
510668	CCR7	A_73_113931	1.0E-4	0.057	chemokine (C-C motif) receptor 7
**TNF family**					
286849	CD40	A_73_110113	0.033	0.36	CD40 molecule, TNF receptor superfamily member 5
574056	TNFSF8	A_73_106418	0.0044	0.24	tumor necrosis factor (ligand) superfamily, member 8
**IL-10 family**					
508044	IL22RA1	A_73_115811	0.0	2.53	interleukin 22 receptor, alpha 1
526285	IL24	A_73_111317	4.0E-4	8.86	interleukin 24

### DEGs between water buffalo and yellow cattle 7 weeks post-infection

After 7 weeks of infection with *S*.*japonicum*, there were 6,353 DEGs between the two hosts. Among these, 4,185 DEGs were also present prior to infection, accounting for 72.9% of the 6,353 DEGs at 7 weeks. Among the 2,168 DEGs that were newly presented after infection, 1,246 were up-regulated, and 922 were down-regulated in the water buffalo group compared with the yellow cattle group.

After 7 weeks post-infection, the GO function analysis of the newly presenting up-regulated genes in water buffalo showed that they were mainly related to the regulation of locomotion, cell motion, transmembrane transporter activity, negative regulation of immune system process, responses to stimuli, transport, metabolism, and biological processes (S3 Table). The newly presenting down-regulated genes in water buffalo were mainly correlated with T cell selection, responses to endogenous stimuli, the positive regulation of developmental processes, and immune system processes, such as immune system development, developmental maturation, etc. (S4 Table). Further pathway analysis of these newly present DEGs between the two natural hosts after 7 weeks of infection showed that they are focused on the following important pathways: natural killer cell-mediated cytotoxicity, complement and coagulation cascades, endocytosis, the hematopoietic cell lineage, cytokine–cytokine receptor interactions, the p53 signaling pathway, the MAPK signaling pathway, apoptosis, the Jak-STAT signaling pathway, the Toll-like receptor signaling pathway, purine metabolism, the B cell receptor signaling pathway (S5 Table). These results revealed that, prior to infection, the newly presenting DEGs take part in many immune-related pathways. Additionally, DEGs belonging to some other important pathways, including cytokine–cytokine receptor interactions, apoptosis, purine metabolism (Table 3), were enriched after infection.

**Table 3:** Functional pathways for newly presenting DEGs in water buffalo compared with yellow cattle 7 weeks after infection^a^.

Pathway	New presenting genes included in pathway (g4_vs_g2)
**Natural killer cell mediated cytotoxicity**	
Up-regulated genes	FCGR3, NFATC3, PRKCA
Down-regulated genes	IFNA14L, CD244, FAS, NCR1, PIK3CD
**Complement and coagulation cascades**	
Up-regulated genes	PLAT
Down-regulated genes	C1QA, C2, CD55, PROS1, SERPING1
**Endocytosis**	
Up-regulated genes	MET, NTRK1
Down-regulated genes	ADRB3, CCR5, CHMP2B, FAM125A, TFRC, WWP1
**Hematopoietic cell lineage**	
Up-regulated genes	-
Down-regulated genes	CD14, CD55, IL1B, ITGA2B, TFRC
**Cytokine-cytokine receptor interaction**	
Up-regulated genes	MET,TNFSF13B
Down-regulated genes	IL14L, CCR5, FAS,IL-15,IL1B,IL20RA, TNFSF13
**p53 signaling pathway**	
Up-regulated genes	IGF1
Down-regulated genes	FAS,IGFBP3,SESN2
**MAPK signaling pathway**	
Up-regulated genes	GNG12, NTRK1, PRKCA
Down-regulated genes	CD14, FAS, IL1B, MAP3K4, MAPT,
**Apotosis**	
Up-regulated genes	NTRK1
Down-regulated genes	FAS, IL1B,PIK3CD
**Jak-STAT signaling pathway**	
Up-regulated genes	-
Down-regulated genes	IL14L, IL15, IL20RA, PIK3CD, STAT2
**Toll-like receptor signaling pathway**	
Up-regulated genes	-
Down-regulated genes	IL14L,CD14, IL1B, PIK3CD
**Purine metabolism**	
Up-regulated genes	PDE3B,POLR2C
Down-regulated genes	DCK,GDUOK,ENPP1
**B cell receptor signaling pathway**	
Up-regulated genes	LOC515489, NFATC3
Down-regulated genes	PIK3CD

^a^ In detail find in S6 Table.

### The common DEGs in water buffalo and yellow cattle post-schistosome infection

**Fig 3:**
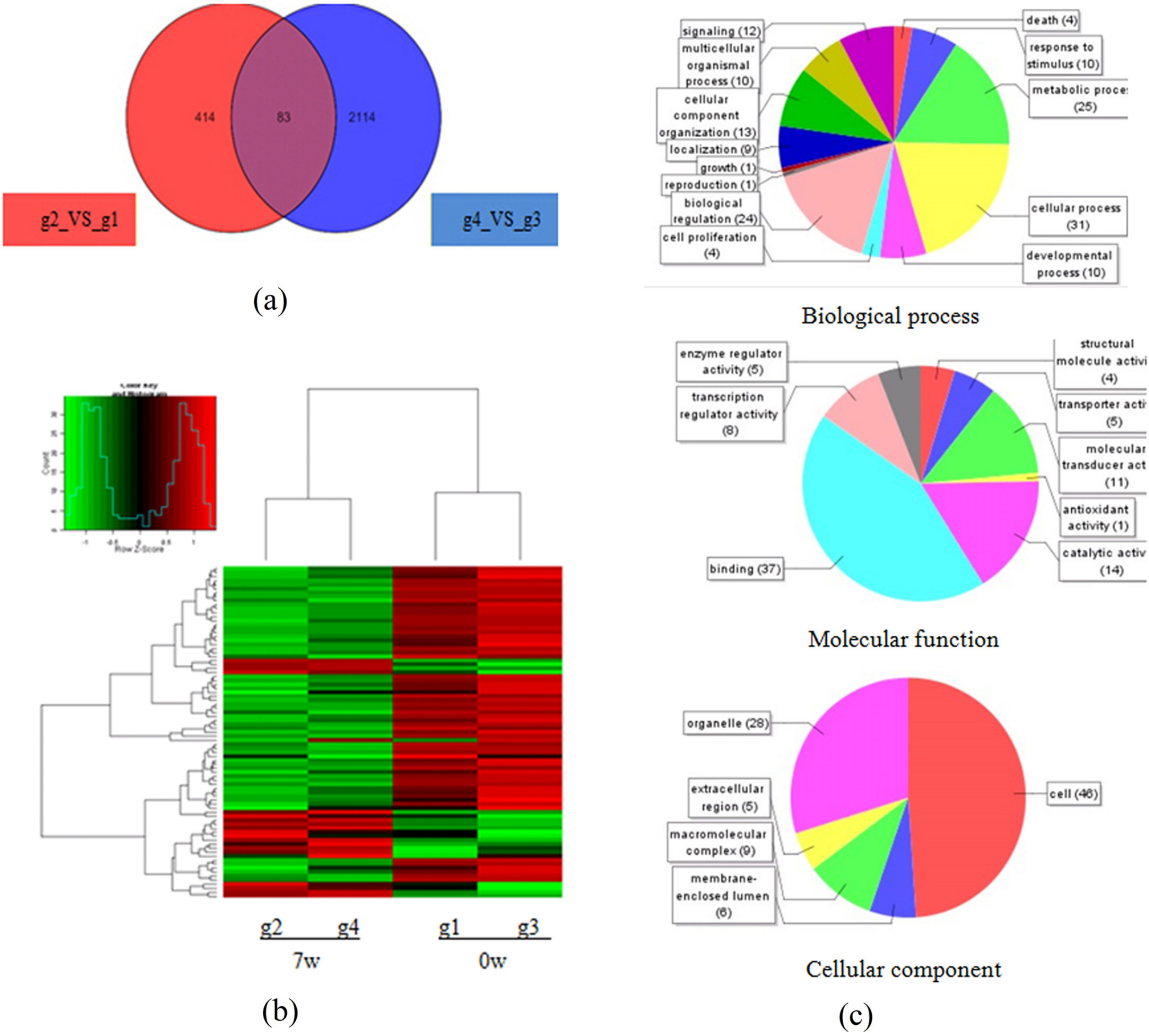
Common DEGs in water buffalo and yellow cattle post-schistosome infection. (a) Common DEGs in two hosts, DEGs both in water buffalo and yellow cattle, 7 weeks post-infection with *S*. *japonicum* compared to pre-infection. (p<0.05, fold change>2); (b) Cluster analysis for common DEGs. (p<0.05, fold change>2); (c) Go Function distribution of common DEGs. (p<0.05).

After 7 weeks of infection with *S*.*japonicum*, only 83 DEGs were found to be in common in water buffalo and yellow cattle (Fig 3A, p<0.05, fold change>2). The cluster analysis of these common genes is shown in Fig 3B. The GO function enrichment analysis showed that the common DEGs are mainly involved in the regulation of fibrinolysis, lipid binding, cell adhesion, nucleic acid binding, transmembrane transporter activity (S6 Table). The GO function analysis for biological process, molecular function, and cellular component is shown in Fig 3C (p<0.05). These common DEGs maybe essential response genes during *S*. *japonicum* infection; a detailed gene list is given in S7 Table.

### DEGs found only in yellow cattle post-schistosome infection

After 7 weeks of infection, 390 DEGs, compared with pre-infection, were found only in yellow cattle, accounting for 78.5% of the total DEGs (497) in yellow cattle. The enrichment analysis of the KEGG signaling pathway for these DEGs showed that they are associated with neuroactive ligand–receptor interactions, cytokine–cytokine receptor interactions, glycosphingolipid biosynthesis, chemokine signaling pathways (S8 Table).

### DEGs found only in water buffalo post-schistosome infection

Compared with yellow cattle, the less-susceptible host water buffalo showed many more DEGs after 7 weeks of infection with *S*.*japonicum* (2,197 versus 497). At the same time, the majority of the DEGs were different in each host; 2,114 were unique to water buffalo, while 390 were unique to yellow cattle.

The enrichment analysis of the KEGG signaling pathway for those DEGs found only in water buffalo showed that they are mainly involved in metabolic pathways, T cell receptor signaling pathways, histidine metabolism, vascular smooth muscle contraction, base excision repair, primary immunodeficiency, leukocyte transendothelial migration, natural killer cell-mediated cytotoxicity, the neurotrophin signaling pathway, the insulin signaling pathway, the Wnt signaling pathway, antigen processing and presentation (S9 Table).

### Validation of mRNA expression by real-time PCR

To validate the microarray transcriptional data, the mRNA expression profiles for a subset of genes from different categories were assessed using quantitative real-time PCR. The selected genes and validation results are presented in Table 4. 22 of the 24 (91.67%) selected genes were validated, which confirmed the results for the directionality of regulation and the fold-change of the microarray experiments.

**Table 4:** Real-time PCR validation of the microarray results^a^.

Accession number	Gene symbol	Gene name	Microarray fold change		Real-time PCR fold change	
DEGs in both water buffalo and yellow cattle post-infection compared to pre-infection			g3_VS_g1	g4_VS_g2	g3_VS_g1	g4_VS_g2
NM_001033617	CTSC	cathepsin C	2.30E-02	3.41E-02	0.66	0.74
AB098980	ND1	NADH dehydrogenase subunit 1	3.66E-03	2.65E-03	0.05	0.09
NM_001098958	CDKN1A	cyclin-dependent kinase inhibitor 1A	23.75	22.66	5.09	1.54
NM_001076517	LY6D	lymphocyte antigen 6 complex, locus D	62.14	56.03	98.30	6.77
NM_001102498	NKAPL	NFKB activating protein-like	35.33	31.67	1.35	3.28
NM_001038611	ZPBP	zonapellucida binding protein	7.79	11.16	19.41	8.28
DEGs in water buffalo post-infection compared to pre-infection			g4_vs_g3		g4_vs_g3	
NM_001081520	LY6G6E	lymphocyte antigen 6 complex, locus G6E	0.23		0.04	
NM_176872	THBS2	thrombospondin 2	0.33		0.07	
DEGs in yellow cattle post-infection compared to pre-infection			g2_vs_g1		g2_vs_g1	
NM_001206292	ZMYM6	PREDICTED: Bos taurus zinc finger, MYM-type 6	2.37		1.29	
NM_001100304	GPR52	GPR52,G protein-coupled receptor 52	2.34		3.55	
NM_174006	CCL2	CCL2,chemokine (C-C motif) ligand 2	0.12		0.16	
NM_001192973	AGMO	Bos taurus alkylglycerolmonooxygenase (AGMO),TMEM195	0.29		0.17	
XM_002694054	TNFRSF8	PREDICTED: Bos taurus Tumor necrosis factor receptor superfamily member 8-like	0.38		0.42	
DEGs common to both yellow catte and water buffalo post-infection compared to pre-infection			g2_vs_g1	g4_vs_g3	g2_vs_g1	g4_vs_g3
NM_001077991	RECQL	RecQ protein-like (DNA helicase Q1-like)	2.26	2.02	2.41	1.12
NM_001014956	NFYA	NFYA,nuclear transcription factor Y, alpha	2.16	2.17	1.39	1.67
NM_174452	ROCK2	Rho-associated, coiled-coil containing protein kinase 2	2.15	2.75	1.97	2.45
DEGs in water buffalo compared to yellow cattle at 7w post-infection			g4_vs_g2		g4_vs_g2	
NM_001080247	JKAMP	JNK1/MAPK8-associated membrane protein	2.62		1.36	
NM_001034492	C2	Bos taurus complement component 2	9.17E-02		0.006	
NM_175703	EBD	Bos taurus defensin, beta 1	0.11		0.41	
NM_174093	IL1B	interleukin 1, beta	0.18		0.25	
NM_173925	IL8	interleukin 8	0.19		0.21	
NM_174008	CD14	CD14 molecule	0.32		0.74	

^a^ The primer sequences used for the validation experiment are provided in S10 Table.

## Discussion

Helminth parasites are generally well-adapted to some hosts, but certain genotypes of their natural host organism are less susceptible to infection, i.e., the water buffalo is less susceptible to *S*. *japonicum* than yellow cattle. However, the mechanism underlying this difference in susceptibility remains unknown. Resistance to infection and the immune clearance of *S*.*japonicum* do not rest on a single molecular mechanism of killing, but rather on the orchestration of multiple pathways that disable and degrade parasites, leading to their expulsion [28, 29]. Both innate and adaptive immuno-mechanisms for helminth infection finely govern host susceptibility. Studies have shown that type 2 immunity protects the host from helminth infections. CD4^+^ T cells can drive a suite of type 2 anti-parasite mechanisms, including class-switched antibodies, activated leukocytes, and innate defensive molecules; the coordination effects of these multiple pathways disable, degrade and wipe out the parasites, leading to their destruction or expulsion [29, 30].

In our previous studies, water buffalo and yellow cattle were sacrificed 7 weeks post-infection with *S*.*japonicum*, at which time their livers showed many histopathological differences; the parasites derived from yellow cattle showed significantly more worm recovery, better worm development, and a different microscopic morphology compared with those derived from water buffalo [12, 17]. Further analysis of the genes responsible for the differences in susceptibility between water buffalo and yellow cattle was performed in this study. Domestic cattle are artificially grouped into 2 species, *Bos taurus* and *Bos indicus*. *Indicus cattle* are the predominant breed in China. The first taurine genome was sequenced in 2009 [19], while the indicine genome was sequenced more recently in 2012 [31]. Comparative analysis of these genomes showed that both breeds shared high similarity at the nucleotide level in all autosomes and the X chromosome, covering 97% of taurine protein coding genes [19, 31]. As water buffalo and yellow cattle are both members of the Bovidae family, they are closely related, and the vast amount of cattle genomic resources might serve as shortcuts for further advances genome science and biotechnology in this species [32]. In 2014, the water buffalo genome research group also declared that the bovine genome could be utilized in association studies [33]. In this paper, a *Bos taurus* genome-wide gene chip was used to analyze and compare the overall gene expression profiles of peripheral blood from water buffalo and yellow cattle, both pre- and post infection with *S*.*japonicum*. The results revealed that prior to schistosome infection, most of the DEGs between water buffalo and yellow cattle were mainly related to immune system function, including the B cell receptor signaling pathway, the T cell receptor signaling pathway, the Toll-like receptor signaling pathway, hematopoietic cell lineage, natural killer cell-mediated cytotoxicity (Table 1). Prior to infection, many genes associated with the immune response/system were expressed at lower levels in the less-susceptible water buffalo, which exhibited less pathological damage and fewer worms developing to a mature state. When the animals were challenged with *S*. *japonicum* for 7 weeks, some other genes of molecules correlated with metabolism, apoptosis, and signal transduction were found to be differentially expressed, and immune-related genes constituted the majority of the DEGs. Genome research on schistosomes has shown that many molecules of schistosomes are similar to those of their hosts. Thus, the molecules of schistosomes could act as receptors for the host’s signals, thereby enabling their growth and development [34,35]. Our findings are consistent with a report on an immunodeficient mouse model infected with *S*. *mansoni* [14]. This study revealed that in immunodeficient hosts, parasites failed to receive appropriate signals from the host immune system, resulting in the appearance of attenuated forms that prolonged the survival of host and parasite. Subsequent studies suggest that the type 2 response driven by CD4^+^ T cells during pre-patent infections of immunocompetent hosts is exploited by schistosomes to complete their development toward reproductively mature adult parasites [16]. In our previous study, the percentage of CD4^+^ T cells in water buffalo was significantly lower than that in yellow cattle [12]; and in this study, the expression levels of some immuno-associated genes in water buffalo were also lower than in yellow cattle, which supports the above observation that an appropriate level of immuno-stimulation from the host is necessary for the development and survival of the worms in their hosts.

Prior to infection, DEGs in the IL-10 family were expressed at higher levels in water buffalo compared with yellow cattle (Table 2). IL-10 family cytokines emerged before the adaptive immune response, and these cytokines elicit diverse host defense mechanism, especially from epithelial cells. IL-10 family cytokines are essential factors for maintaining the integrity and homeostasis of tissue epithelial layers [36]. During infection, IL-10 family cytokines can promote innate immune responses from tissue epithelia to limit the damage from infection. To establish a successful infection, schistosomes first need to penetrate the host by breaking through the skin. The IL-10 family cytokines IL-22R and IL-24 were over-expressed by 2.53- and 8.86-fold, respectively, in water buffalo, which indicated that the skin layer defense for schistosomes in water buffalo might be more effective than that in yellow cattle.

After 7 weeks of infection with *S*.*japonicum*, there were 6,353 DEGs between the two hosts; 2,168 of these DEGs were newly presented after infection. Further analysis showed that the newly presenting DEGs between the two hosts were mainly associated with the innate immune system, immune regulation, hematopoietic cells, thep53 signaling pathway, purine metabolism, etc., and most of the DEGs were under-expressed in water buffalo compared with yellow cattle. The pathway enrichment analysis showed that the DEGs constituted similar immune-related pathways, both pre- and post-infection, between the two hosts, which suggests that native differences between the two hosts are likely to be the key factors that affect the establishment and maintenance of *S*. *japonicum* infections.

After infection, water buffalo and yellow cattle showed different changes compared with pre-infection, and most DEGs were uniquely found only in yellow cattle or water buffalo; only 83 DEGs were observed in both hosts. These 83 common DEGs maybe the essential response genes during *S*.*japonicum* infection, and GO function analysis showed that the highest two GO functions were associated with the regulation of fibrinolysis (S6 Table), which is the special pathological damage resulting from schistosome infections. The unique DEGs (390) in yellow cattle were mainly enriched in pathways of cytokine–cytokine receptor interactions, neuroactive ligand–receptor interactions, chemokine signaling pathways, etc., which is consistent with the phenomenon that more intense inflammation occurred in yellow cattle than in water buffalo after infection [12]. The unique DEGs (2,114) in water buffalo were mainly associated with immune-related pathways, such as T cell receptor signaling, primary immunodeficiency, antigen processing and presentation, etc., which again indicates that immune factors likely contribute to *S*. *japonicum* resistance in water buffalo.

This study was the first to compare and analyze the overall gene expression profiles in the peripheral blood from water buffalo and yellow cattle, both pre- and post-infection with *S*.*japonicum*. It will increase our understanding of the relationship between parasites and the different susceptibilities of natural hosts.

## Supporting Information

S1 Table Function pathway of up-regulated genes in water buffalo compared to yellow cattle pre-infection. (DOC)

S2 Table Function pathway of under-regulated genes in water buffalo compared to yellow cattle pre- infection. (DOC)

S3 Table Enrichment analysis of Go function for new presenting over-expressed genes in water buffalo compared to those in yellow cattle 7weeks after infection. (DOC)

S4 Table Enrichment analysis of Go function for new presenting under-expressed genes in water buffalo compared to those in yellow cattle 7weeks after infection. (DOC)

S5 Table Some function pathway for new presenting DEGs between water buffalo and yellow cattle 7weeks after infection. (DOC)

S6 Table Enrichment analysis of Go function for DEGs both in water buffalo and yellow cattle post-infection with *Sj* 7w compared to pre-infection. (DOC)

S7 Table List of some DEGs both in water buffalo and yellow cattle post-infection with *Sj* 7w compared to pre-infection. (DOC)

S8 Table Enrichment analysis of KEGG pathway for DEGs unique to yellow cattle 7 weeks post-inection with *S*. *japonicum* compared with pre-infection. (DOC)

S9 Table Enrichment analysis of KEGG pathway for DEGs unique to water buffalo 7 weeks post-infection with *S*. *japonicum* compared with pre-infection. (DOC)

S10 Table Primers in real-time PCR validation for microarray results. (DOC)
