# Supplementary material for: The Differential Expression of Immune Genes between Water Buffalo and Yellow Cattle Determines Species-Specific Susceptibility to Schistosoma japonicum Infection
Source: PLoS One. 2015 Jun 30;10(6):e0130344. doi: 10.1371/journal.pone.0130344 (PMC4488319; doi:10.1371/journal.pone.0130344)
Supplement: S1 Table — (DOC) [file pone.0130344.s001.doc]

**S1 Table. Function pathway of up-regulated genes in water buffalo compared to yellow cattle pre-infection.**

| **Gene ID** | **Symbol** | **Probe ID** | **p value** | **g3_vs_g1**  **fold change** | **Gene name** |
| --- | --- | --- | --- | --- | --- |
| **Tight junction** | | | | | |
| 524770 | ACTN1 | A_73_111323 | 0.0108 | 2.24 | actinin, alpha 1 |
| 506545 | CLDN10 | A_73_106401 | 0.0039 | 44.34 | claudin 10 |
| 508445 | HCLS1 | A_73_120474 | 0.0248 | 2.59 | hematopoietic cell-specific Lyn substrate 1 |
| 536863 | MPDZ | A_73_113861 | 0.0022 | 10.78 | multiple PDZ domain protein |
| 519251 | MRK | A_73_112212 | 0.0086 | 2.86 |  |
| 281338 | MYH3 | A_73_117196 | 0.0059 | 10.49 | myosin, heavy chain 3, skeletal muscle, embryonic |
| 408020 | MYH4 | A_73_116984 | 2.0E-4 | 18.68 | Myosin heavy chain-2B-like protein Fragment |
| 535321 | PPP2R1A | A_73_106939 | 0.010 | 2.04 | protein phosphatase 2, regulatory subunit A, alpha |
| 282325 | PRKCB | A_73_113451 | 0.0051 | 2.08 | Protein kinase C, beta |
| 528478 | PRKCI | A_73_118034 | 0.027 | 2.32 | Protein kinase C, iota |
| **TGF-beta signaling pathway** | | | | | |
| 280760 | DCN | A_73_111861 | 0.0028 | 4.07 | Decorin |
| 286859 | GDF7 | A_73_113829 | 0.038 | 2.09 | Growth differentiation factor 7 |
| 534018 | LEFTY2 | A_73_107797 | 0.0035 | 2.87 | left-right determination factor 2 |
| 535321 | PPP2R1A | A_73_106939 | 0.010 | 2.04 | protein phosphatase 2, regulatory subunit A, alpha |
