# Supplementary material for: The Differential Expression of Immune Genes between Water Buffalo and Yellow Cattle Determines Species-Specific Susceptibility to Schistosoma japonicum Infection
Source: PLoS One. 2015 Jun 30;10(6):e0130344. doi: 10.1371/journal.pone.0130344 (PMC4488319; doi:10.1371/journal.pone.0130344)
Supplement: S2 Table — (DOC) [file pone.0130344.s002.doc]

**S2 Table. Function pathway of under-regulated genes in water buffalo compared to yellow cattle pre- infection.**

| **Gene ID** | **Symbol** | **Probe ID** | **p value** | **g3_vs_g1**  **fold change** | **Gene name** |
| --- | --- | --- | --- | --- | --- |
| **p53 signaling pathway** | | | | | |
| 327679 | CCNB1 | A_73_115691 | 1.0E-4 | 0.18 | cyclin B1 |
| 538436 | CCNE2 | A_73_120096 | 0.0249 | 0.27 | cyclin E2 |
| 281061 | CDK1 | A_73_107660 | 0.0021 | 0.23 | cyclin-dependent kinase 1 |
| 510618 | CDK4 | A_73_108052 | 0.0128 | 0.46 | cyclin-dependent kinase 4 |
| 515914 | SESN3 | A_73_119882 | 1.0E-4 | 0.19 | sestrin 3 |
| **B cell receptor signaling pathway** | | | | | |
| 353510 | IFITM1 | A_73_120243 | 0.047 | 0.45 | interferon induced transmembrane protein 1 (9-27) |
| 281855 | IKBKG | A_73_108677 | 0.012 | 0.48 | inhibitor of kappa light polypeptide gene enhancer in B-cells, kinase gamma |
| 280831 | JUN | A_73_120007 | 0.036 | 0.49 | jun proto-oncogene |
| 534996 | LYN | A_73_105751 | 0.0026 | 0.45 | v-yes-1 Yamaguchi sarcoma viral related oncogene homolog |
| **T cell receptor signaling pathway** | | | | | |
| 510618 | CDK4 | A_73_108052 | 0.013 | 0.46 | cyclin-dependent kinase 4 |
| 281855 | IKBKG | A_73_108677 | 0.012 | 0.48 | inhibitor of kappa light polypeptide gene enhancer in B-cells, kinase gamma |
| 280831 | JUN | A_73_120007 | 0.036 | 0.49 | jun proto-oncogene |
| 535327 | MAPK13 | A_73_105507 | 8.0E-4 | 5.76E-3 | mitogen-activated protein kinase 13 |
| **Hematopoietic cell lineage** | | | | | |
| 281861 | IL2RA | A_73_109472 | 0.0042 | 0.07 | interleukin 2 receptor, alpha |
| 404154 | IL4R | A_73_112133 | 0.0014 | 0.37 | interleukin 4 receptor |
| 282882 | ITGA4 | A_73_107613 | 0.0014 | 0.097 | integrin, alpha 4 (antigen CD49D, alpha 4 subunit of VLA-4 receptor) |
| **Toll-like receptor signaling pathway** | | | | | |
| 286849 | CD40 | A_73_110113 | 0.033 | 0.36 | CD40 molecule, TNF receptor superfamily member 5 |
| 407131 | CD80 | A_73_110255 | 9.0E-4 | 0.049 | CD80 molecule |
| 282257 | IFNAR1 | A_73_116078 | 0.035 | 0.47 | interferon (alpha, beta and omega) receptor 1 |
| 281855 | IKBKG | A_73_108677 | 0.012 | 0.48 | inhibitor of kappa light polypeptide gene enhancer in B-cells, kinase gamma |
| 280831 | JUN | A_73_120007 | 0.036 | 0.49 | jun proto-oncogene |
| 512242 | LBP | A_73_104583 | 0.022 | 0.38 | lipopolysaccharide binding protein |
| 535327 | MAPK13 | A_73_105507 | 8.0E-4 | 5.76E-3 | mitogen-activated protein kinase 13 |
| **Natural killer cell mediated cytotoxicity** | | | | | |
| 508619 | IFNGR1 | A_73_115260 | 3.0E-4 | 1.44E-3 | interferon gamma receptor 1 |
| 514889 | IFNGR2 | A_73_107789 | 0.0 | 5.39E-3 | interferon gamma receptor 2 |
| 407173 | JSP.1 | A_73_112038 | 0.037 | 0.088 | MHC Class I JSP.1 |
| 281731 | GZMB | A_73_112113 | 0.032 | 4.11 | granzyme B (granzyme 2, cytotoxic T-lymphocyte-associated serine esterase 1) |
| 282257 | IFNAR1 | A_73_116078 | 0.035 | 0.24 | interferon (alpha, beta and omega) receptor 1 |
| 613356 | SH2D1A | A_73_117272 | 0.0073 | 0.48 | SH2 domain containing 1A |
