# Supplementary material for: The Differential Expression of Immune Genes between Water Buffalo and Yellow Cattle Determines Species-Specific Susceptibility to Schistosoma japonicum Infection
Source: PLoS One. 2015 Jun 30;10(6):e0130344. doi: 10.1371/journal.pone.0130344 (PMC4488319; doi:10.1371/journal.pone.0130344)
Supplement: S3 Table — (DOC) [file pone.0130344.s003.doc]

**S3 Table. Enrichment analysis of Go function for new presenting over-expressed genes in water buffalo compared to those in yellow cattle 7weeks after infection.**

| **GO Id** | **Name** | [**Hits**](javascript:void(0);) | | [**Total**](javascript:void(0);) | [**Percent**](javascript:void(0);) | [**Enrichment test p value**](javascript:void(0);) | [**q value**](javascript:void(0);) |
| --- | --- | --- | --- | --- | --- | --- | --- |
| GO:0040012 | regulation of locomotion | | [4](http://sas.ebioservice.com/bioinfoplug_molnetgotree.showgene.do?acc=GO:0040012&name=regulation+of+locomotion&recordid=70451233&loginid=BH11042) | 71 | 5.63% | 0.03 | 1 |
| GO:0017140 | lipoic acid synthase activity | | [1](http://sas.ebioservice.com/bioinfoplug_molnetgotree.showgene.do?acc=GO:0017140&name=lipoic+acid+synthase+activity&recordid=70451233&loginid=BH11042) | 1 | 100.00% | 0.031 | 1 |
| GO:0070283 | radical SAM enzyme activity | | [1](http://sas.ebioservice.com/bioinfoplug_molnetgotree.showgene.do?acc=GO:0070283&name=radical+SAM+enzyme+activity&recordid=70451233&loginid=BH11042) | 1 | 100.00% | 0.031 | 1 |
| GO:0045177 | apical part of cell | | [3](http://sas.ebioservice.com/bioinfoplug_molnetgotree.showgene.do?acc=GO:0045177&name=apical+part+of+cell&recordid=70451233&loginid=BH11042) | 52 | 5.77% | 0.055 | 1 |
| GO:0031254 | trailing edge | | [1](http://sas.ebioservice.com/bioinfoplug_molnetgotree.showgene.do?acc=GO:0031254&name=trailing+edge&recordid=70451233&loginid=BH11042) | 3 | 33.33% | 0.061 | 1 |
| GO:0006928 | cell motion | | [6](http://sas.ebioservice.com/bioinfoplug_molnetgotree.showgene.do?acc=GO:0006928&name=cell+motion&recordid=70451233&loginid=BH11042) | 178 | 3.37% | 0.070 | 1 |
| GO:0051674 | localization of cell | | [6](http://sas.ebioservice.com/bioinfoplug_molnetgotree.showgene.do?acc=GO:0051674&name=localization+of+cell&recordid=70451233&loginid=BH11042) | 178 | 3.37% | 0.070 | 1 |
| GO:0002683 | negative regulation of immune system process | | [2](http://sas.ebioservice.com/bioinfoplug_molnetgotree.showgene.do?acc=GO:0002683&name=negative+regulation+of+immune+system+process&recordid=70451233&loginid=BH11042) | 26 | 7.69% | 0.071 | 1 |
| GO:0022892 | substrate-specific transporter activity | | [14](http://sas.ebioservice.com/bioinfoplug_molnetgotree.showgene.do?acc=GO:0022892&name=substrate-specific+transporter+activity&recordid=70451233&loginid=BH11042) | 578 | 2.42% | 0.082 | 1 |
| GO:0048585 | negative regulation of response to stimulus | | [2](http://sas.ebioservice.com/bioinfoplug_molnetgotree.showgene.do?acc=GO:0048585&name=negative+regulation+of+response+to+stimulus&recordid=70451233&loginid=BH11042) | 29 | 6.90% | 0.085 | 1 |
| GO:0040011 | locomotion | | [6](http://sas.ebioservice.com/bioinfoplug_molnetgotree.showgene.do?acc=GO:0040011&name=locomotion&recordid=70451233&loginid=BH11042) | 192 | 3.13% | 0.092 | 1 |
| GO:0022857 | transmembrane transporter activity | | [13](http://sas.ebioservice.com/bioinfoplug_molnetgotree.showgene.do?acc=GO:0022857&name=transmembrane+transporter+activity&recordid=70451233&loginid=BH11042) | 543 | 2.39% | 0.098 | 1 |
| GO:0050900 | leukocyte migration | | [2](http://sas.ebioservice.com/bioinfoplug_molnetgotree.showgene.do?acc=GO:0050900&name=leukocyte+migration&recordid=70451233&loginid=BH11042) | 32 | 6.25% | 0.099 | 1 |
| GO:0007163 | establishment or maintenance of cell polarity | | [1](http://sas.ebioservice.com/bioinfoplug_molnetgotree.showgene.do?acc=GO:0007163&name=establishment+or+maintenance+of+cell+polarity&recordid=70451233&loginid=BH11042) | 6 | 16.67% | 0.11 | 1 |
| GO:0048870 | cell motility | | [5](http://sas.ebioservice.com/bioinfoplug_molnetgotree.showgene.do?acc=GO:0048870&name=cell+motility&recordid=70451233&loginid=BH11042) | 156 | 3.21% | 0.11 | 1 |
| GO:0001871 | pattern binding | | [3](http://sas.ebioservice.com/bioinfoplug_molnetgotree.showgene.do?acc=GO:0001871&name=pattern+binding&recordid=70451233&loginid=BH11042) | 73 | 4.11% | 0.12 | 1 |
| GO:0010576 | metalloenzyme regulator activity | | [1](http://sas.ebioservice.com/bioinfoplug_molnetgotree.showgene.do?acc=GO:0010576&name=metalloenzyme+regulator+activity&recordid=70451233&loginid=BH11042) | 7 | 14.29% | 0.12 | 1 |
| GO:0019827 | stem cell maintenance | | [1](http://sas.ebioservice.com/bioinfoplug_molnetgotree.showgene.do?acc=GO:0019827&name=stem+cell+maintenance&recordid=70451233&loginid=BH11042) | 8 | 12.50% | 0.13 | 1 |
| GO:0030534 | adult behavior | | [2](http://sas.ebioservice.com/bioinfoplug_molnetgotree.showgene.do?acc=GO:0030534&name=adult+behavior&recordid=70451233&loginid=BH11042) | 41 | 4.88% | 0.15 | 1 |
| GO:0051051 | negative regulation of transport | | [2](http://sas.ebioservice.com/bioinfoplug_molnetgotree.showgene.do?acc=GO:0051051&name=negative+regulation+of+transport&recordid=70451233&loginid=BH11042) | 45 | 4.44% | 0.17 | 1 |
| GO:0060090 | molecular adaptor activity | | [1](http://sas.ebioservice.com/bioinfoplug_molnetgotree.showgene.do?acc=GO:0060090&name=molecular+adaptor+activity&recordid=70451233&loginid=BH11042) | 11 | 9.09% | 0.17 | 1 |
| GO:0005215 | transporter activity | | [15](http://sas.ebioservice.com/bioinfoplug_molnetgotree.showgene.do?acc=GO:0005215&name=transporter+activity&recordid=70451233&loginid=BH11042) | 719 | 2.09% | 0.18 | 1 |
| GO:0009892 | negative regulation of metabolic process | | [6](http://sas.ebioservice.com/bioinfoplug_molnetgotree.showgene.do?acc=GO:0009892&name=negative+regulation+of+metabolic+process&recordid=70451233&loginid=BH11042) | 235 | 2.55% | 0.18 | 1 |
| GO:0048519 | negative regulation of biological process | | [12](http://sas.ebioservice.com/bioinfoplug_molnetgotree.showgene.do?acc=GO:0048519&name=negative+regulation+of+biological+process&recordid=70451233&loginid=BH11042) | 556 | 2.16% | 0.18 | 1 |
