# Supplementary material for: The Differential Expression of Immune Genes between Water Buffalo and Yellow Cattle Determines Species-Specific Susceptibility to Schistosoma japonicum Infection
Source: PLoS One. 2015 Jun 30;10(6):e0130344. doi: 10.1371/journal.pone.0130344 (PMC4488319; doi:10.1371/journal.pone.0130344)
Supplement: S4 Table — (DOC) [file pone.0130344.s004.doc]

**S4 Table. Enrichment analysis of Go function for new presenting under-expressed genes in water buffalo compared to those in yellow cattle 7weeks after infection.**

| **GO Id** | **Name** | [**Hits**](javascript:void(0);) | [**Total**](javascript:void(0);) | [**Percent**](javascript:void(0);) | [**Enrichment test p value**](javascript:void(0);) | [**q value**](javascript:void(0);) |
| --- | --- | --- | --- | --- | --- | --- |
| GO:0045058 | T cell selection | [2](http://sas.ebioservice.com/bioinfoplug_molnetgotree.showgene.do?acc=GO:0045058&name=T+cell+selection&recordid=70451234&loginid=BH11042) | 6 | 33.33% | 0.0048 | 0.51 |
| GO:0009719 | response to endogenous stimulus | [4](http://sas.ebioservice.com/bioinfoplug_molnetgotree.showgene.do?acc=GO:0009719&name=response+to+endogenous+stimulus&recordid=70451234&loginid=BH11042) | 56 | 7.14% | 0.0086 | 0.51 |
| GO:0002682 | regulation of immune system process | [7](http://sas.ebioservice.com/bioinfoplug_molnetgotree.showgene.do?acc=GO:0002682&name=regulation+of+immune+system+process&recordid=70451234&loginid=BH11042) | 170 | 4.12% | 0.01 | 0.51 |
| GO:0002252 | immune effector process | [5](http://sas.ebioservice.com/bioinfoplug_molnetgotree.showgene.do?acc=GO:0002252&name=immune+effector+process&recordid=70451234&loginid=BH11042) | 95 | 5.26% | 0.011 | 0.51 |
| GO:0019825 | oxygen binding | [2](http://sas.ebioservice.com/bioinfoplug_molnetgotree.showgene.do?acc=GO:0019825&name=oxygen+binding&recordid=70451234&loginid=BH11042) | 13 | 15.38% | 0.017 | 0.55 |
| GO:0051094 | positive regulation of developmental process | [7](http://sas.ebioservice.com/bioinfoplug_molnetgotree.showgene.do?acc=GO:0051094&name=positive+regulation+of+developmental+process&recordid=70451234&loginid=BH11042) | 195 | 3.59% | 0.019 | 0.55 |
| GO:0045177 | apical part of cell | [3](http://sas.ebioservice.com/bioinfoplug_molnetgotree.showgene.do?acc=GO:0045177&name=apical+part+of+cell&recordid=70451234&loginid=BH11042) | 52 | 5.77% | 0.038 | 0.69 |
| GO:0030246 | carbohydrate binding | [5](http://sas.ebioservice.com/bioinfoplug_molnetgotree.showgene.do?acc=GO:0030246&name=carbohydrate+binding&recordid=70451234&loginid=BH11042) | 133 | 3.76% | 0.039 | 0.69 |
| GO:0048469 | cell maturation | [2](http://sas.ebioservice.com/bioinfoplug_molnetgotree.showgene.do?acc=GO:0048469&name=cell+maturation&recordid=70451234&loginid=BH11042) | 25 | 8.00% | 0.051 | 0.69 |
| GO:0002376 | immune system process | [11](http://sas.ebioservice.com/bioinfoplug_molnetgotree.showgene.do?acc=GO:0002376&name=immune+system+process&recordid=70451234&loginid=BH11042) | 458 | 2.40% | 0.052 | 0.69 |
| GO:0048589 | developmental growth | [2](http://sas.ebioservice.com/bioinfoplug_molnetgotree.showgene.do?acc=GO:0048589&name=developmental+growth&recordid=70451234&loginid=BH11042) | 27 | 7.41% | 0.058 | 0.69 |
| GO:0002520 | immune system development | [5](http://sas.ebioservice.com/bioinfoplug_molnetgotree.showgene.do?acc=GO:0002520&name=immune+system+development&recordid=70451234&loginid=BH11042) | 151 | 3.31% | 0.060 | 0.69 |
| GO:0048584 | positive regulation of response to stimulus | [4](http://sas.ebioservice.com/bioinfoplug_molnetgotree.showgene.do?acc=GO:0048584&name=positive+regulation+of+response+to+stimulus&recordid=70451234&loginid=BH11042) | 107 | 3.74% | 0.063 | 0.69 |
| GO:0006955 | immune response | [8](http://sas.ebioservice.com/bioinfoplug_molnetgotree.showgene.do?acc=GO:0006955&name=immune+response&recordid=70451234&loginid=BH11042) | 307 | 2.61% | 0.063 | 0.69 |
| GO:0004857 | enzyme inhibitor activity | [5](http://sas.ebioservice.com/bioinfoplug_molnetgotree.showgene.do?acc=GO:0004857&name=enzyme+inhibitor+activity&recordid=70451234&loginid=BH11042) | 156 | 3.21% | 0.066 | 0.69 |
| GO:0002684 | positive regulation of immune system process | [4](http://sas.ebioservice.com/bioinfoplug_molnetgotree.showgene.do?acc=GO:0002684&name=positive+regulation+of+immune+system+process&recordid=70451234&loginid=BH11042) | 115 | 3.48% | 0.077 | 0.69 |
| GO:0051239 | regulation of multicellular organismal process | [8](http://sas.ebioservice.com/bioinfoplug_molnetgotree.showgene.do?acc=GO:0051239&name=regulation+of+multicellular+organismal+process&recordid=70451234&loginid=BH11042) | 326 | 2.45% | 0.082 | 0.69 |
| GO:0021700 | developmental maturation | [2](http://sas.ebioservice.com/bioinfoplug_molnetgotree.showgene.do?acc=GO:0021700&name=developmental+maturation&recordid=70451234&loginid=BH11042) | 34 | 5.88% | 0.084 | 0.69 |
| GO:0043028 | caspase regulator activity | [1](http://sas.ebioservice.com/bioinfoplug_molnetgotree.showgene.do?acc=GO:0043028&name=caspase+regulator+activity&recordid=70451234&loginid=BH11042) | 6 | 16.67% | 0.091 | 0.69 |
| GO:0045730 | respiratory burst | [1](http://sas.ebioservice.com/bioinfoplug_molnetgotree.showgene.do?acc=GO:0045730&name=respiratory+burst&recordid=70451234&loginid=BH11042) | 6 | 16.67% | 0.091 | 0.69 |
