# Supplementary material for: The Differential Expression of Immune Genes between Water Buffalo and Yellow Cattle Determines Species-Specific Susceptibility to Schistosoma japonicum Infection
Source: PLoS One. 2015 Jun 30;10(6):e0130344. doi: 10.1371/journal.pone.0130344 (PMC4488319; doi:10.1371/journal.pone.0130344)
Supplement: S5 Table — (DOC) [file pone.0130344.s005.doc]

**S5 Table. Some function pathway for new presenting DEGs between water buffalo and yellow cattle 7weeks after infection.**

| **Gene ID** | **Symbol** | **Probe ID** | **p value** | | | **g4_vs_g2**  **fold change** | **Gene name** |
| --- | --- | --- | --- | --- | --- | --- | --- |
| **Natural killer cell mediated cytotoxicity** | | | | | | | |
| 517108 | IFNA14L | A_73_103660 | 2.0E-4 | | | 0.29 | interferon, alpha 14-like |
| 513468 | CD244 | A_73_110545 | 0.0377 | | | 0.30 | CD244 molecule, natural killer cell receptor 2B4 |
| 282488 | FAS | A_73_105727 | 0.04 | | | 0.48 | Fas (TNF receptor superfamily, member 6) |
| 281766 | FCGR3 | A_73_102350 | 0.0324 | | | 2.88 | Fc fragment of IgG, low affinity IIIa, receptor (CD16a) |
| 369024 | NCR1 | A_73_100699 | 0.0281 | | | 0.40 | natural cytotoxicity triggering receptor 1 |
| 541127 | NFATC3 | A_73_103491 | 0.0024 | | | 2.80 | nuclear factor of activated T-cells, cytoplasmic, calcineurin-dependent 3 |
| 504531 | PIK3CD | A_73_105764 | 0.0097 | | | 0.50 | phosphoinositide-3-kinase, catalytic, delta polypeptide |
| 282001 | PRKCA | A_73_110824 | 0.0070 | | | 2.40 | protein kinase C, alpha |
| **Complement and coagulation cascades** | | | | | | | |
| 534961 | C1QA | A_73_118275 | 0.0062 | | | 0.50 | complement component 1, q subcomponent, A chain |
| 515440 | C2 | A_73_101342 | 0.016 | | | 0.092 | complement component 2 |
| 518609 | CD55 | A_73_116775 | 0.0017 | | | 0.37 | CD55 molecule, decay accelerating factor for complement (Cromer blood group) |
| 281407 | PLAT | A_73_112170 | 0.0025 | | | 7.56 | plasminogen activator, tissue |
| 282006 | PROS1 | A_73_120060 | 0.045 | | | 0.057 | protein S (alpha) |
| 281035 | SERPING1 | A_73_109408 | 0.0092 | | | 0.28 | serpin peptidase inhibitor, clade G (C1 inhibitor), member 1 |
| **Endocytosis** | | | | | | | |
| 281606 | ADRB3 | A_73_121299 | 0.019 | | | 0.28 | adrenergic, beta-3-, receptor |
| 497017 | CCR5 | A_73_115370 | 0.0067 | | | 0.46 | chemokine (C-C motif) receptor 5 |
| 615954 | CHMP2B | A_73_110306 | 0.012 | | | 0.44 | charged multivesicular body protein 2B |
| 507199 | FAM125A | A_73_111906 | 3.0E-4 | | | 0.47 | family with sequence similarity 125, member A |
| 280855 | MET | A_73_116287 | 0.035 | | | 2.25 | met proto-oncogene (hepatocyte growth factor receptor) |
| 353111 | NTRK1 | A_73_103532 | 0.022 | | | 4.50 | neurotrophic tyrosine kinase, receptor, type 1 |
| 504698 | TFRC | A_73_113842 | 0.014 | | | 0.19 | transferrin receptor (p90, CD71) |
| 513789 | WWP1 | A_73_109254 | 3.0E-4 | | | 0.11 | WW domain containing E3 ubiquitin protein ligase 1 |
| 613428 | ZFYVE9 | A_73_100893 | 0.0042 | | | 0.20 |  |
| **Hematopoietic cell lineage** | | | | | | | |
| 281048 | CD14 | A_73_116002 | 0.0041 | | | 0.32 | CD14 molecule |
| 518609 | CD55 | A_73_116775 | 0.0017 | | | 0.37 | CD55 molecule, decay accelerating factor for complement (Cromer blood group) |
| 281251 | IL1B | A_73_110556 | 0.017 | | | 0.18 | interleukin 1, beta |
| 515011 | ITGA2B | A_73_120486 | 0.030 | | | 0.46 | integrin, alpha 2b (platelet glycoprotein IIb of IIb/IIIa complex, antigen CD41) |
| 504698 | TFRC | A_73_113842 | 0.014 | | | 0.19 | transferrin receptor (p90, CD71) |
| **Cytokine-cytokine receptor interaction** | | | | | | | |
| 517108 | LOC517108 | A_73_103660 | 2.0E-4 | | | 0.29 | interferon, alpha 14-like |
| 497017 | CCR5 | A_73_115370 | 0.0067 | | | 0.46 | chemokine (C-C motif) receptor 5 |
| 282488 | FAS | A_73_105727 | 0.04 | | | 0.48 | Fas (TNF receptor superfamily, member 6) |
| 281248 | IL15 | A_73_101953 | 0.047 | | | 0.47 | interleukin 15 |
| 281251 | IL1B | A_73_110556 | 0.017 | | | 0.18 | interleukin 1, beta |
| 509038 | IL20RA | A_73_100950 | 0.037 | | | 0.42 | interleukin 20 receptor, alpha |
| 280855 | MET | A_73_116287 | 0.035 | | | 2.25 | met proto-oncogene (hepatocyte growth factor receptor) |
| 538567 | TNFSF13 | A_73_116859 | 0.016 | | | 0.47 | tumor necrosis factor (ligand) superfamily, member 13 |
| 504507 | TNFSF13B | A_73_112644 | 0.0016 | | | 2.28 | tumor necrosis factor (ligand) superfamily, member 13b |
| **p53 signaling pathway** | | | | | | | |
| 282488 | FAS | A_73_105727 | 0.04 | | | 0.48 | Fas (TNF receptor superfamily, member 6) |
| 281239 | IGF1 | A_73_108570 | 0.0037 | | | 5.12 | insulin-like growth factor 1 (somatomedin C) |
| 282261 | IGFBP3 | A_73_120953 | 0.0032 | | | 0.25 | insulin-like growth factor binding protein 3 |
| 509863 | SESN2 | A_73_116162 | 0.0094 | | | 0.36 | sestrin 2 |
| **MAPK signaling pathway** | | | | | | | |
| 281048 | CD14 | A_73_116002 | 0.0041 | | | 0.32 | CD14 molecule |
| 282488 | FAS | A_73_105727 | 0.04 | | | 0.48 | Fas (TNF receptor superfamily, member 6) |
| 286850 | GNG12 | A_73_121257 | 0.0107 | | | 2.018 | guanine nucleotide binding protein (G protein), gamma 12 |
| 281251 | IL1B | A_73_110556 | 0.017 | | | 0.18 | interleukin 1, beta |
| 511779 | MAP3K4 | A_73_119119 | 0.0050 | | | 0.45 | mitogen-activated protein kinase kinase kinase 4 |
| 281296 | MAPT | A_73_111960 | 0.024 | | | 0.44 | microtubule-associated protein tau |
| 353111 | NTRK1 | A_73_103532 | 0.022 | | | 4.50 | neurotrophic tyrosine kinase, receptor, type 1 |
| 282001 | PRKCA | A_73_110824 | 0.0070 | | | 2.39 | protein kinase C, alpha |
| **Apoptosis** | | | | | | | |
| 282488 | FAS | A_73_105727 | 0.04 | | | 0.48 | Fas (TNF receptor superfamily, member 6) |
| 281251 | IL1B | A_73_110556 | 0.017 | | | 0.18 | interleukin 1, beta |
| 353111 | NTRK1 | A_73_103532 | 0.022 | | | 4.50 | neurotrophic tyrosine kinase, receptor, type 1 |
| 504531 | PIK3CD | A_73_105764 | 0.010 | | | 0.50 | phosphoinositide-3-kinase, catalytic, delta polypeptide |
| **Jak-STAT signaling pathway** | | | | | | | |
| 517108 | LOC517108 | A_73_103660 | 2.0E-4 | | | 0.29 | interferon, alpha 14-like |
| 281248 | IL15 | A_73_101953 | 0.047 | | | 0.47 | interleukin 15 |
| 509038 | IL20RA | A_73_100950 | 0.037 | | | 0.42 | interleukin 20 receptor, alpha |
| 504531 | PIK3CD | A_73_105764 | 0.0097 | | | 0.50 | phosphoinositide-3-kinase, catalytic, delta polypeptide |
| 511023 | STAT2 | A_73_108484 | 0.035 | | | 0.36 | signal transducer and activator of transcription 2, 113kDa |
| **Toll-like receptor signaling pathway** | | | | | | | |
| 517108 | LOC517108 | A_73_103660 | 2.0E-4 | | | 0.29 | interferon, alpha 14-like |
| 281048 | CD14 | A_73_116002 | 0.0041 | | | 0.32 | CD14 molecule |
| 281251 | IL1B | A_73_110556 | 0.017 | | | 0.18 | interleukin 1, beta |
| 504531 | PIK3CD | A_73_105764 | 0.0097 | | | 0.50 | phosphoinositide-3-kinase, catalytic, delta polypeptide |
| **Purine metabolism** | | | | | | | |
| 530642 | DCK | A_73_113269 | | 0.017 | 0.30 | | deoxycytidine kinase |
| 509834 | DGUOK | A_73_106682 | | 1.0E-4 | 0.47 | | deoxyguanosine kinase |
| 615535 | ENPP1 | A_73_109405 | | 0.035 | 0.31 | | ectonucleotide pyrophosphatase/phosphodiesterase 1 |
| 533323 | PDE3B | A_73_104539 | | 0.0033 | 3.94 | | phosphodiesterase 3B, cGMP-inhibited |
| 504452 | POLR2C | A_73_107498 | | 0.017 | 2.07 | | polymerase (RNA) II (DNA directed) polypeptide C, 33kDa |
| **B cell receptor signaling pathway** | | | | | | | |
| 515489 | LOC515489 | A_73_109474 | 0.0012 | | | 2.25 | translocase of outer mitochondrial membrane 5 homolog |
| 541127 | NFATC3 | A_73_103491 | 0.0024 | | | 2.80 | nuclear factor of activated T-cells, cytoplasmic, calcineurin-dependent 3 |
| 504531 | PIK3CD | A_73_105764 | 0.0097 | | | 0.50 | phosphoinositide-3-kinase, catalytic, delta polypeptide |
