# Supplementary material for: The Differential Expression of Immune Genes between Water Buffalo and Yellow Cattle Determines Species-Specific Susceptibility to Schistosoma japonicum Infection
Source: PLoS One. 2015 Jun 30;10(6):e0130344. doi: 10.1371/journal.pone.0130344 (PMC4488319; doi:10.1371/journal.pone.0130344)
Supplement: S6 Table — (DOC) [file pone.0130344.s006.doc]

**S6 Table. Enrichment analysis of Go function for DEGs both in water buffalo and yellow cattle post-infection with *Sj*** 7w compared to pre-infection.

| **GO Id** | **Name** | [**Hits**](javascript:void(0);) | [**Total**](javascript:void(0);) | [**Percent**](javascript:void(0);) | [**Enrichment test p value**](javascript:void(0);) | [**q value**](javascript:void(0);) |
| --- | --- | --- | --- | --- | --- | --- |
| GO:0051918 | negative regulation of fibrinolysis | [1](http://sas.ebioservice.com/bioinfoplug_molnetgotree.showgene.do?acc=GO:0051918&name=negative+regulation+of+fibrinolysis&recordid=62521630&loginid=BH11042) | 3 | 33.33% | 0.0047 | 0.20 |
| GO:0051917 | regulation of fibrinolysis | [1](http://sas.ebioservice.com/bioinfoplug_molnetgotree.showgene.do?acc=GO:0051917&name=regulation+of+fibrinolysis&recordid=62521630&loginid=BH11042) | 4 | 25.00% | 0.0059 | 0.20 |
| GO:0050817 | coagulation | [1](http://sas.ebioservice.com/bioinfoplug_molnetgotree.showgene.do?acc=GO:0050817&name=coagulation&recordid=62521630&loginid=BH11042) | 57 | 1.75% | 0.067 | 0.87 |
| GO:0050878 | regulation of body fluid levels | [1](http://sas.ebioservice.com/bioinfoplug_molnetgotree.showgene.do?acc=GO:0050878&name=regulation+of+body+fluid+levels&recordid=62521630&loginid=BH11042) | 64 | 1.56% | 0.074 | 0.87 |
| GO:0051241 | negative regulation of multicellular organismal process | [1](http://sas.ebioservice.com/bioinfoplug_molnetgotree.showgene.do?acc=GO:0051241&name=negative+regulation+of+multicellular+organismal+process&recordid=62521630&loginid=BH11042) | 68 | 1.47% | 0.079 | 0.87 |
| GO:0004857 | enzyme inhibitor activity | [1](http://sas.ebioservice.com/bioinfoplug_molnetgotree.showgene.do?acc=GO:0004857&name=enzyme+inhibitor+activity&recordid=62521630&loginid=BH11042) | 156 | 0.64% | 0.17 | 1.0 |
| GO:0048583 | regulation of response to stimulus | [1](http://sas.ebioservice.com/bioinfoplug_molnetgotree.showgene.do?acc=GO:0048583&name=regulation+of+response+to+stimulus&recordid=62521630&loginid=BH11042) | 171 | 0.58% | 0.19 | 1.0 |
| GO:0048037 | cofactor binding | [1](http://sas.ebioservice.com/bioinfoplug_molnetgotree.showgene.do?acc=GO:0048037&name=cofactor+binding&recordid=62521630&loginid=BH11042) | 176 | 0.57% | 0.19 | 1.0 |
| GO:0008289 | lipid binding | [1](http://sas.ebioservice.com/bioinfoplug_molnetgotree.showgene.do?acc=GO:0008289&name=lipid+binding&recordid=62521630&loginid=BH11042) | 221 | 0.45% | 0.23 | 1.0 |
| GO:0031982 | vesicle | [1](http://sas.ebioservice.com/bioinfoplug_molnetgotree.showgene.do?acc=GO:0031982&name=vesicle&recordid=62521630&loginid=BH11042) | 245 | 0.41% | 0.26 | 1.0 |
| GO:0009605 | response to external stimulus | [1](http://sas.ebioservice.com/bioinfoplug_molnetgotree.showgene.do?acc=GO:0009605&name=response+to+external+stimulus&recordid=62521630&loginid=BH11042) | 294 | 0.34% | 0.30 | 1.0 |
| GO:0007155 | cell adhesion | [1](http://sas.ebioservice.com/bioinfoplug_molnetgotree.showgene.do?acc=GO:0007155&name=cell+adhesion&recordid=62521630&loginid=BH11042) | 325 | 0.31% | 0.32 | 1.0 |
| GO:0022610 | biological adhesion | [1](http://sas.ebioservice.com/bioinfoplug_molnetgotree.showgene.do?acc=GO:0022610&name=biological+adhesion&recordid=62521630&loginid=BH11042) | 325 | 0.31% | 0.32 | 1.0 |
| GO:0007155 | cell adhesion | [1](http://sas.ebioservice.com/bioinfoplug_molnetgotree.showgene.do?acc=GO:0007155&name=cell+adhesion&recordid=62521630&loginid=BH11042) | 325 | 0.31% | 0.32 | 1.0 |
| GO:0051239 | regulation of multicellular organismal process | [1](http://sas.ebioservice.com/bioinfoplug_molnetgotree.showgene.do?acc=GO:0051239&name=regulation+of+multicellular+organismal+process&recordid=62521630&loginid=BH11042) | 326 | 0.31% | 0.36 | 1.0 |
| GO:0003676 | nucleic acid binding | [3](http://sas.ebioservice.com/bioinfoplug_molnetgotree.showgene.do?acc=GO:0003676&name=nucleic+acid+binding&recordid=62521630&loginid=BH11042) | 1755 | 0.17% | 0.35 | 1.0 |
| GO:0051179 | localization | [3](http://sas.ebioservice.com/bioinfoplug_molnetgotree.showgene.do?acc=GO:0051179&name=localization&recordid=62521630&loginid=BH11042) | 1765 | 0.17% | 0.35 | 1.0 |
| GO:0003700 | transcription factor activity | [1](http://sas.ebioservice.com/bioinfoplug_molnetgotree.showgene.do?acc=GO:0003700&name=transcription+factor+activity&recordid=62521630&loginid=BH11042) | 379 | 0.26% | 0.37 | 1.0 |
| GO:0055085 | transmembrane transport | [1](http://sas.ebioservice.com/bioinfoplug_molnetgotree.showgene.do?acc=GO:0055085&name=transmembrane+transport&recordid=62521630&loginid=BH11042) | 392 | 0.26% | 0.38 | 1.0 |
| GO:0030234 | enzyme regulator activity | [1](http://sas.ebioservice.com/bioinfoplug_molnetgotree.showgene.do?acc=GO:0030234&name=enzyme+regulator+activity&recordid=62521630&loginid=BH11042) | 403 | 0.25% | 0.39 | 1.0 |
| GO:0022857 | transmembrane transporter activity | [1](http://sas.ebioservice.com/bioinfoplug_molnetgotree.showgene.do?acc=GO:0022857&name=transmembrane+transporter+activity&recordid=62521630&loginid=BH11042) | 543 | 0.18% | 0.48 | 1.0 |
| GO:0043167 | ion binding | [3](http://sas.ebioservice.com/bioinfoplug_molnetgotree.showgene.do?acc=GO:0043167&name=ion+binding&recordid=62521630&loginid=BH11042) | 2163 | 0.14% | 0.49 | 1.0 |
| GO:0048519 | negative regulation of biological process | [1](http://sas.ebioservice.com/bioinfoplug_molnetgotree.showgene.do?acc=GO:0048519&name=negative+regulation+of+biological+process&recordid=62521630&loginid=BH11042) | 556 | 0.18% | 0.49 | 1.0 |
