# Supplementary material for: The Differential Expression of Immune Genes between Water Buffalo and Yellow Cattle Determines Species-Specific Susceptibility to Schistosoma japonicum Infection
Source: PLoS One. 2015 Jun 30;10(6):e0130344. doi: 10.1371/journal.pone.0130344 (PMC4488319; doi:10.1371/journal.pone.0130344)
Supplement: S7 Table — (DOC) [file pone.0130344.s007.doc]

**S7 Table. List of some DEGs both in water buffalo and yellow cattle post-infection with *Sj*** 7w compared to pre-infection.

| **Probe Name** | **Genbank**  **Accession** | **Gene Name** | **Chromosome Number** | **TIGRID** | **g2_VS_g1**  **fold change** | **g4_VS_g3**  **fold change** |
| --- | --- | --- | --- | --- | --- | --- |
| A_73_P264751 | NM_001077991 | RecQ protein-like (DNA helicase Q1-like) | chr5 | TC310246 | 2.26 | 2.02 |
| A_73_P382031 | NM_001014956 | nuclear transcription factor Y, alpha | chr23 | TC360096 | 2.16 | 2.17 |
| A_73_P035041 | NM_174452 | Rho-associated, coiled-coil containing protein kinase 2 | chr11 | TC371770 | 2.15 | 2.75 |
| A_73_118773 | XM_582657 | zinc finger CCCH-type containing 6 | chr11 | TC348170 | 2.09 | 2.96 |
| A_73_114765 | NM_001110532 | BCL2-associated athanogene 4 | chr27 | TC368805 | 2.09 | 5.83 |
| A_73_P044496 | NM_001105460 | vacuolar protein sorting 54 homolog (S. cerevisiae) | chr11 | TC368700 | 2.06 | 3.30 |
| A_73_P044141 | NM_174236 | A kinase (PRKA) anchor protein 5 | chr10 | TC302586 | 2.04 | 3.95 |
| A_73_106659 | XM_599034 | hypothetical LOC520784 | chr4 | TC313900 | 2.00 | 3.97 |
| A_73_P355491 | NM_001077978 | cysteine conjugate-beta lyase, cytoplasmic | chr11 | TC350794 | 0.50 | 0.43 |
| A_73_117758 | XM_604851 | zinc finger protein 496 | chr7 | -NA- | 0.50 | 0.41 |
| A_73_P079066 | XM_586913 | similar to UPF0172 protein C14orf122 | chr10 | TC341170 | 0.50 | 0.38 |
| A_73_P193927 | NM_001083753 | SH3 domain containing ring finger 2 | chr7 | TC350657 | 0.50 | 0.39 |
| A_73_101790 | NM_001192193 | T-box 4 | chr19 | -NA- | 0.49 | 0.41 |
| A_73_114808 | NM_173919 | histidine-rich glycoprotein | chr1 | TC302091 | 0.49 | 0.43 |
| A_73_P353981 | XM_867976 | ankyrin repeat and sterile alpha motif domain containing 3 | chr25 | TC350212 | 0.49 | 0.42 |
| A_73_P374416 | XM_866405 | pleckstrin homology domain containing, family G (with RhoGef domain) member 2 | chr18 | TC357563 | 0.49 | 0.48 |
| A_73_P300666 | XM_593953 | peroxidasin homolog (Drosophila) | - | TC327805 | 0.49 | 0.23 |
| A_73_P488784 | XM_002687448 | similar to olfactory receptor MOR114-1 | chr5 | -NA- | 0.49 | 0.47 |
| A_73_117093 | XM_589215 | zinc finger protein 648 | chr16 | -NA- | 0.49 | 0.43 |
| A_73_P117941 | DV786248 | ribosomal protein L3-like | chr5 | -NA- | 0.48 | 0.49 |
| A_73_111583 | NM_001046138 | ras homolog gene family, member C | chr3 | TC375577 | 0.48 | 0.49 |
| A_73_P104301 | NM_174542 | gamma-aminobutyric acid (GABA) A receptor, alpha 3 | chrX | TC302059 | 0.48 | 0.33 |
| A_73_P323621 | NM_001080234 | zinc finger, CCHC domain containing 3 | chr13 | TC338175 | 0.47 | 0.48 |
| A_73_119578 | NM_181038 | acyl-CoA binding domain containing 5 | chr13 | TC302023 | 0.47 | 0.46 |
| A_73_P075121 | XM_002693125 | pleckstrin homology-like domain, family B, member 1 | chr15 | TC319227 | 0.47 | 0.42 |
| A_73_P321026 | NM_001075800 | E2F-associated phosphoprotein | chr21 | TC337102 | 0.47 | 0.36 |
| A_73_P154537 | DV789388 | high-mobility group box 1 | - | -NA- | 0.47 | 0.49 |
| A_73_109596 | XR_083725 | fizzy-like | chr7 | TC348822 | 0.47 | 0.43 |
| A_73_P385741 | NM_001192763 | zinc finger, DHHC-type containing 5 | chr15 | TC361332 | 0.46 | 0.48 |
| A_73_109103 | NM_001144093 | OTU domain containing 7B | chr3 | TC349798 | 0.46 | 0.48 |
| A_73_P491998 | XM_002698229 | hypothetical protein LOC100140410 | chr25 | -NA- | 0.44 | 0.44 |
| A_73_P478233 | XM_599206 | sphingomyelin synthase 2 | chr6 | TC389609 | 0.44 | 0.45 |
| A_73_P056901 | XM_002687993 | similar to membrane-associated DHHC26 zinc finger protein | - | -NA- | 0.44 | 0.35 |
| A_73_P290591 | NM_001075178 | interleukin 17 receptor C | chr22 | TC323013 | 0.44 | 0.47 |
| A_73_P253436 | NM_001046063 | serpin peptidase inhibitor, clade H (heat shock protein 47), member 1, (collagen binding protein 1) | chr15 | TC303928 | 0.44 | 0.32 |
| A_73_P330186 | NM_001192931 | cadherin, EGF LAG seven-pass G-type receptor 2 (flamingo homolog, Drosophila) | chr3 | TC340849 | 0.44 | 0.41 |
| A_73_P065456 | NM_001077139 | ring finger protein 112 | chr19 | -NA- | 0.44 | 0.50 |
| A_73_P046666 | NM_001078105 | cysteine/tyrosine-rich 1 | chr1 | TC326756 | 0.43 | 0.45 |
| A_73_P124706 | CF766020 | stimulated by retinoic acid 13 homolog (mouse) | - | -NA- | 0.43 | 0.44 |
| A_73_103871 | NM_001075830 | matrix-remodelling associated 8 | chr16 | TC316979 | 0.43 | 0.36 |
| A_73_P349176 | NM_001075761 | heart and neural crest derivatives expressed 1 | chr7 | TC348332 | 0.43 | 0.33 |
| A_73_115028 | NM_174723 | Rh family, B glycoprotein (gene/pseudogene) | chr3 | TC302366 | 0.43 | 0.25 |
| A_73_P107706 | EU548078 | solute carrier family 5 (sodium iodide symporter), member 5 | chr7 | TC339121 | 0.42 | 0.41 |
| A_73_P269201 | NM_001075673 | prenylcysteine oxidase 1 like | chr7 | TC312464 | 0.41 | 0.36 |
| A_73_P092141 | XM_002685938 | olfactory receptor, family 6, subfamily Y, member 1 | chr3 | -NA- | 0.41 | 0.39 |
| A_73_P056071 | XM_001255695 | similar to olfactory receptor Olr1522 | chr19 | -NA- | 0.41 | 0.47 |
| A_73_P166712 | XM_588109 | proline rich Gla (G-carboxyglutamic acid) 3 (transmembrane) | chrX | TC345490 | 0.41 | 0.36 |
| A_73_P123061 | NM_001192213 | heme binding protein 2 | chr9 | TC311022 | 0.40 | 0.35 |
| A_73_P039156 | NM_001076483 | angiopoietin 4 | chr13 | TC345414 | 0.40 | 0.38 |
| A_73_P490543 | XM_002698021 | otoancorin | chr25 | -NA- | 0.39 | 0.38 |
| A_73_P092311 | NM_001192474 | cholinergic receptor, nicotinic, beta 2 (neuronal) | chr3 | -NA- | 0.39 | 0.43 |
| A_73_P113936 | XM_599153 | forkhead box A2 | chr13 | TC325857 | 0.38 | 0.40 |
| A_73_P486493 | XM_001253224 | keratin associated protein 15-1 | chr1 | -NA- | 0.38 | 0.33 |
| A_73_P084816 | XM_002689066 | similar to rCG58596 | chr7 | -NA- | 0.38 | 0.27 |
| A_73_P079116 | NM_001192889 | solute carrier family 7 (amino acid transporter, L-type), member 8 | chr10 | TC349986 | 0.37 | 0.48 |
| A_73_118130 | NM_001077065 | solute carrier family 9 (sodium/hydrogen exchanger), member 3 regulator 2 | chr25 | TC306044 | 0.37 | 0.39 |
| A_73_P091621 | XM_872665 | nuclear receptor interacting protein 2 | chr5 | TC348785 | 0.35 | 0.30 |
| A_73_116629 | NM_001103298 | hypothetical protein LOC615263 | chr2 | TC342940 | 0.35 | 0.36 |
| A_73_P296416 | NM_001098385 | keratin | chr5 | TC325777 | 0.34 | 0.33 |
| A_73_P103616 | BC109934 | hypothetical LOC782599 | chr22 | -NA- | 0.26 | 0.30 |
