# Supplementary material for: The Differential Expression of Immune Genes between Water Buffalo and Yellow Cattle Determines Species-Specific Susceptibility to Schistosoma japonicum Infection
Source: PLoS One. 2015 Jun 30;10(6):e0130344. doi: 10.1371/journal.pone.0130344 (PMC4488319; doi:10.1371/journal.pone.0130344)
Supplement: S8 Table — (DOC) [file pone.0130344.s008.doc]

**S8 Table. Enrichment analysis of KEGG pathway for DEGs unique to yellow cattle 7 weeks post-inection with *S. japonicum*** compared with pre-infection.

| **Pathway Name** | [**Hits**](javascript:void(0);) | [**Total**](javascript:void(0);) | [**Percent**](javascript:void(0);) | [**Enrichment test p value**](javascript:void(0);) | [**q value**](javascript:void(0);) |
| --- | --- | --- | --- | --- | --- |
| [Neuroactive ligand-receptor interaction](http://sas.ebioservice.com/bioinfoplug_molnetpathwaygene.showframe.do?projectid=defaultdb&db=kegg&recordid=62521633&loginid=BH11042&pathwayid=bta04080&gene=517040,GABBR2;529656,GRM6;504216,NPY;617879,GIPR;613414,TRH&pathwayname=Neuroactive+ligand-receptor+interaction+-+Bos+taurus+(cow)&pictureext=png) | 5 | 305 | 1.64% | 8.00E-04 | 0.0035 |
| [Cytokine-cytokine receptor interaction](http://sas.ebioservice.com/bioinfoplug_molnetpathwaygene.showframe.do?projectid=defaultdb&db=kegg&recordid=62521633&loginid=BH11042&pathwayid=bta04060&gene=281043,CCL2;286859,GDF7;338031,FLT4&pathwayname=Cytokine-cytokine+receptor+interaction+-+Bos+taurus+(cow)&pictureext=png) | 3 | 249 | 1.20% | 0.022 | 0.046 |
| [Glycosphingolipid biosynthesis - globo series](http://sas.ebioservice.com/bioinfoplug_molnetpathwaygene.showframe.do?projectid=defaultdb&db=kegg&recordid=62521633&loginid=BH11042&pathwayid=bta00603&gene=281175,FUT2&pathwayname=Glycosphingolipid+biosynthesis+-+globo+series+-+Bos+taurus+(cow)&pictureext=png) | 1 | 15 | 6.67% | 0.037 | 0.047 |
| [Glycosphingolipid biosynthesis - lacto and neolacto series](http://sas.ebioservice.com/bioinfoplug_molnetpathwaygene.showframe.do?projectid=defaultdb&db=kegg&recordid=62521633&loginid=BH11042&pathwayid=bta00601&gene=281175,FUT2&pathwayname=Glycosphingolipid+biosynthesis+-+lacto+and+neolacto+series+-+Bos+taurus+(cow)&pictureext=png) | 1 | 25 | 4.00% | 0.059 | 0.047 |
| [Chemokine signaling pathway](http://sas.ebioservice.com/bioinfoplug_molnetpathwaygene.showframe.do?projectid=defaultdb&db=kegg&recordid=62521633&loginid=BH11042&pathwayid=bta04062&gene=513340,GNB3;281043,CCL2&pathwayname=Chemokine+signaling+pathway+-+Bos+taurus+(cow)&pictureext=png) | 2 | 179 | 1.12% | 0.068 | 0.047 |
| [Tyrosine metabolism](http://sas.ebioservice.com/bioinfoplug_molnetpathwaygene.showframe.do?projectid=defaultdb&db=kegg&recordid=62521633&loginid=BH11042&pathwayid=bta00350&gene=280761,DCT&pathwayname=Tyrosine+metabolism+-+Bos+taurus+(cow)&pictureext=png) | 1 | 32 | 3.13% | 0.075 | 0.047 |
| [Taste transduction](http://sas.ebioservice.com/bioinfoplug_molnetpathwaygene.showframe.do?projectid=defaultdb&db=kegg&recordid=62521633&loginid=BH11042&pathwayid=bta04742&gene=513340,GNB3&pathwayname=Taste+transduction+-+Bos+taurus+(cow)&pictureext=png) | 1 | 34 | 2.94% | 0.079 | 0.047 |
| [mTOR signaling pathway](http://sas.ebioservice.com/bioinfoplug_molnetpathwaygene.showframe.do?projectid=defaultdb&db=kegg&recordid=62521633&loginid=BH11042&pathwayid=bta04150&gene=614570,EIF4E1B&pathwayname=mTOR+signaling+pathway+-+Bos+taurus+(cow)&pictureext=png) | 1 | 53 | 1.89% | 0.12 | 0.047 |
| [NOD-like receptor signaling pathway](http://sas.ebioservice.com/bioinfoplug_molnetpathwaygene.showframe.do?projectid=defaultdb&db=kegg&recordid=62521633&loginid=BH11042&pathwayid=bta04621&gene=281043,CCL2&pathwayname=NOD-like+receptor+signaling+pathway+-+Bos+taurus+(cow)&pictureext=png) | 1 | 57 | 1.75% | 0.13 | 0.047 |
| [MAPK signaling pathway](http://sas.ebioservice.com/bioinfoplug_molnetpathwaygene.showframe.do?projectid=defaultdb&db=kegg&recordid=62521633&loginid=BH11042&pathwayid=bta04010&gene=615727,DUSP8;521616,CACNG6&pathwayname=MAPK+signaling+pathway+-+Bos+taurus+(cow)&pictureext=png) | 2 | 265 | 0.75% | 0.13 | 0.047 |
| [Arrhythmogenic right ventricular cardiomyopathy (ARVC)](http://sas.ebioservice.com/bioinfoplug_molnetpathwaygene.showframe.do?projectid=defaultdb&db=kegg&recordid=62521633&loginid=BH11042&pathwayid=bta05412&gene=521616,CACNG6&pathwayname=Arrhythmogenic+right+ventricular+cardiomyopathy+(ARVC)+-+Bos+taurus+(cow)&pictureext=png) | 1 | 66 | 1.52% | 0.15 | 0.047 |
