# Supplementary material for: The Differential Expression of Immune Genes between Water Buffalo and Yellow Cattle Determines Species-Specific Susceptibility to Schistosoma japonicum Infection
Source: PLoS One. 2015 Jun 30;10(6):e0130344. doi: 10.1371/journal.pone.0130344 (PMC4488319; doi:10.1371/journal.pone.0130344)
Supplement: S9 Table — (DOC) [file pone.0130344.s009.doc]

**S9 Table. Enrichment analysis of KEGG pathway for DEGs unique to water buffalo 7 weeks post-infection with *S. japonicum*** compared with pre-infection.

| **Name** | [**Hits**](javascript:void(0);) | [**Total**](javascript:void(0);) | [**Percent**](javascript:void(0);) | [**Enrichment test p value**](javascript:void(0);) | [**q value**](javascript:void(0);) |
| --- | --- | --- | --- | --- | --- |
| [Metabolic pathways](http://sas.ebioservice.com/bioinfoplug_molnetpathwaygene.showframe.do?projectid=defaultdb&db=kegg&recordid=62521629&loginid=BH11042&pathwayid=bta01100&gene=353301,MECR;541272,IDS;515270,GAMT;280871,MUT;281230,HPSE;281119,DNMT1;509519,LDHAL6B;280960,XDH;506349,INPP5E;282200,COX6A2;515950,HDC;539606,PYCR1;281987,PLCG1;3283886,ND4;444860,ST3GAL6;282854,FUT4&pathwayname=Metabolic+pathways+-+Bos+taurus+(cow)&pictureext=png) | 16 | 1065 | 1.50% | 0.0045 | 0.020 |
| [T cell receptor signaling pathway](http://sas.ebioservice.com/bioinfoplug_molnetpathwaygene.showframe.do?projectid=defaultdb&db=kegg&recordid=62521629&loginid=BH11042&pathwayid=bta04660&gene=541127,NFATC3;504733,TEC;508890,LCK;282387,CD40LG&pathwayname=T+cell+receptor+signaling+pathway+-+Bos+taurus+(cow)&pictureext=png) | 4 | 106 | 3.77% | 0.0079 | 0.020 |
| [Histidine metabolism](http://sas.ebioservice.com/bioinfoplug_molnetpathwaygene.showframe.do?projectid=defaultdb&db=kegg&recordid=62521629&loginid=BH11042&pathwayid=bta00340&gene=515950,HDC;613413,HNMT&pathwayname=Histidine+metabolism+-+Bos+taurus+(cow)&pictureext=png) | 2 | 20 | 10.00% | 0.011 | 0.020 |
| [Vascular smooth muscle contraction](http://sas.ebioservice.com/bioinfoplug_molnetpathwaygene.showframe.do?projectid=defaultdb&db=kegg&recordid=62521629&loginid=BH11042&pathwayid=bta04270&gene=282041,ROCK2;505901,PRKCQ;281341,MYL6;281788,GNA11&pathwayname=Vascular+smooth+muscle+contraction+-+Bos+taurus+(cow)&pictureext=png) | 4 | 122 | 3.28% | 0.013 | 0.020 |
| [Glycosaminoglycan degradation](http://sas.ebioservice.com/bioinfoplug_molnetpathwaygene.showframe.do?projectid=defaultdb&db=kegg&recordid=62521629&loginid=BH11042&pathwayid=bta00531&gene=541272,IDS;281230,HPSE&pathwayname=Glycosaminoglycan+degradation+-+Bos+taurus+(cow)&pictureext=png) | 2 | 24 | 8.33% | 0.015 | 0.020 |
| [Glycosphingolipid biosynthesis series](http://sas.ebioservice.com/bioinfoplug_molnetpathwaygene.showframe.do?projectid=defaultdb&db=kegg&recordid=62521629&loginid=BH11042&pathwayid=bta00601&gene=444860,ST3GAL6;282854,FUT4&pathwayname=Glycosphingolipid+biosynthesis+-+lacto+and+neolacto+series+-+Bos+taurus+(cow)&pictureext=png) | 2 | 25 | 8.00% | 0.016 | 0.020 |
| [Base excision repair](http://sas.ebioservice.com/bioinfoplug_molnetpathwaygene.showframe.do?projectid=defaultdb&db=kegg&recordid=62521629&loginid=BH11042&pathwayid=bta03410&gene=515499,PCNA;615359,PARP4&pathwayname=Base+excision+repair+-+Bos+taurus+(cow)&pictureext=png) | 2 | 31 | 6.45% | 0.023 | 0.020 |
| [Propanoate metabolism](http://sas.ebioservice.com/bioinfoplug_molnetpathwaygene.showframe.do?projectid=defaultdb&db=kegg&recordid=62521629&loginid=BH11042&pathwayid=bta00640&gene=280871,MUT;509519,LDHAL6B&pathwayname=Propanoate+metabolism+-+Bos+taurus+(cow)&pictureext=png) | 2 | 32 | 6.25% | 0.024 | 0.020 |
| [Primary immunodeficiency](http://sas.ebioservice.com/bioinfoplug_molnetpathwaygene.showframe.do?projectid=defaultdb&db=kegg&recordid=62521629&loginid=BH11042&pathwayid=bta05340&gene=508890,LCK;282387,CD40LG&pathwayname=Primary+immunodeficiency+-+Bos+taurus+(cow)&pictureext=png) | 2 | 32 | 6.25% | 0.024 | 0.020 |
| [Cysteine and methionine metabolism](http://sas.ebioservice.com/bioinfoplug_molnetpathwaygene.showframe.do?projectid=defaultdb&db=kegg&recordid=62521629&loginid=BH11042&pathwayid=bta00270&gene=281119,DNMT1;509519,LDHAL6B&pathwayname=Cysteine+and+methionine+metabolism+-+Bos+taurus+(cow)&pictureext=png) | 2 | 33 | 6.06% | 0.026 | 0.020 |
| [Fc gamma R-mediated phagocytosis](http://sas.ebioservice.com/bioinfoplug_molnetpathwaygene.showframe.do?projectid=defaultdb&db=kegg&recordid=62521629&loginid=BH11042&pathwayid=bta04666&gene=505901,PRKCQ;327705,ASAP1;281987,PLCG1&pathwayname=Fc+gamma+R-mediated+phagocytosis+-+Bos+taurus+(cow)&pictureext=png) | 3 | 88 | 3.41% | 0.027 | 0.020 |
| [Caffeine metabolism](http://sas.ebioservice.com/bioinfoplug_molnetpathwaygene.showframe.do?projectid=defaultdb&db=kegg&recordid=62521629&loginid=BH11042&pathwayid=bta00232&gene=280960,XDH&pathwayname=Caffeine+metabolism+-+Bos+taurus+(cow)&pictureext=png) | 1 | 5 | 20.00% | 0.042 | 0.025 |
| [Leukocyte transendothelial migration](http://sas.ebioservice.com/bioinfoplug_molnetpathwaygene.showframe.do?projectid=defaultdb&db=kegg&recordid=62521629&loginid=BH11042&pathwayid=bta04670&gene=282041,ROCK2;407124,ITGAM;281987,PLCG1&pathwayname=Leukocyte+transendothelial+migration+-+Bos+taurus+(cow)&pictureext=png) | 3 | 115 | 2.61% | 0.052 | 0.025 |
| [Arginine and proline metabolism](http://sas.ebioservice.com/bioinfoplug_molnetpathwaygene.showframe.do?projectid=defaultdb&db=kegg&recordid=62521629&loginid=BH11042&pathwayid=bta00330&gene=515270,GAMT;539606,PYCR1&pathwayname=Arginine+and+proline+metabolism+-+Bos+taurus+(cow)&pictureext=png) | 2 | 52 | 3.85% | 0.056 | 0.025 |
| [mTOR signaling pathway](http://sas.ebioservice.com/bioinfoplug_molnetpathwaygene.showframe.do?projectid=defaultdb&db=kegg&recordid=62521629&loginid=BH11042&pathwayid=bta04150&gene=280829,INS;505850,EIF4B&pathwayname=mTOR+signaling+pathway+-+Bos+taurus+(cow)&pictureext=png) | 2 | 53 | 3.77% | 0.058 | 0.025 |
| [Tight junction](http://sas.ebioservice.com/bioinfoplug_molnetpathwaygene.showframe.do?projectid=defaultdb&db=kegg&recordid=62521629&loginid=BH11042&pathwayid=bta04530&gene=282714,MYH7;514541,RAB13;505901,PRKCQ&pathwayname=Tight+junction+-+Bos+taurus+(cow)&pictureext=png) | 3 | 124 | 2.42% | 0.062 | 0.025 |
| [Inositol phosphate metabolism](http://sas.ebioservice.com/bioinfoplug_molnetpathwaygene.showframe.do?projectid=defaultdb&db=kegg&recordid=62521629&loginid=BH11042&pathwayid=bta00562&gene=506349,INPP5E;281987,PLCG1&pathwayname=Inositol+phosphate+metabolism+-+Bos+taurus+(cow)&pictureext=png) | 2 | 56 | 3.57% | 0.064 | 0.025 |
| [Natural killer cell mediated cytotoxicity](http://sas.ebioservice.com/bioinfoplug_molnetpathwaygene.showframe.do?projectid=defaultdb&db=kegg&recordid=62521629&loginid=BH11042&pathwayid=bta04650&gene=541127,NFATC3;281987,PLCG1;508890,LCK&pathwayname=Natural+killer+cell+mediated+cytotoxicity+-+Bos+taurus+(cow)&pictureext=png) | 3 | 126 | 2.38% | 0.064 | 0.025 |
| [Cell adhesion molecules (CAMs)](http://sas.ebioservice.com/bioinfoplug_molnetpathwaygene.showframe.do?projectid=defaultdb&db=kegg&recordid=62521629&loginid=BH11042&pathwayid=bta04514&gene=281840,ICAM3;407124,ITGAM;282387,CD40LG&pathwayname=Cell+adhesion+molecules+(CAMs)+-+Bos+taurus+(cow)&pictureext=png) | 3 | 129 | 2.33% | 0.068 | 0.025 |
| [Fatty acid elongation in mitochondria](http://sas.ebioservice.com/bioinfoplug_molnetpathwaygene.showframe.do?projectid=defaultdb&db=kegg&recordid=62521629&loginid=BH11042&pathwayid=bta00062&gene=353301,MECR&pathwayname=Fatty+acid+elongation+in+mitochondria+-+Bos+taurus+(cow)&pictureext=png) | 1 | 9 | 11.11% | 0.069 | 0.025 |
| [Neurotrophin signaling pathway](http://sas.ebioservice.com/bioinfoplug_molnetpathwaygene.showframe.do?projectid=defaultdb&db=kegg&recordid=62521629&loginid=BH11042&pathwayid=bta04722&gene=504408,RPS6KA5;281987,PLCG1;506066,SH2B1&pathwayname=Neurotrophin+signaling+pathway+-+Bos+taurus+(cow)&pictureext=png) | 3 | 130 | 2.31% | 0.069 | 0.025 |
| [Cell cycle](http://sas.ebioservice.com/bioinfoplug_molnetpathwaygene.showframe.do?projectid=defaultdb&db=kegg&recordid=62521629&loginid=BH11042&pathwayid=bta04110&gene=281729,SMC3;515499,PCNA;524870,RBL1&pathwayname=Cell+cycle+-+Bos+taurus+(cow)&pictureext=png) | 3 | 131 | 2.29% | 0.070 | 0.025 |
| [Insulin signaling pathway](http://sas.ebioservice.com/bioinfoplug_molnetpathwaygene.showframe.do?projectid=defaultdb&db=kegg&recordid=62521629&loginid=BH11042&pathwayid=bta04910&gene=533323,PDE3B;511961,PRKAG3;280829,INS&pathwayname=Insulin+signaling+pathway+-+Bos+taurus+(cow)&pictureext=png) | 3 | 132 | 2.27% | 0.071 | 0.025 |
| [Viral myocarditis](http://sas.ebioservice.com/bioinfoplug_molnetpathwaygene.showframe.do?projectid=defaultdb&db=kegg&recordid=62521629&loginid=BH11042&pathwayid=bta05416&gene=282714,MYH7;282387,CD40LG&pathwayname=Viral+myocarditis+-+Bos+taurus+(cow)&pictureext=png) | 2 | 65 | 3.08% | 0.082 | 0.027 |
| [Wnt signaling pathway](http://sas.ebioservice.com/bioinfoplug_molnetpathwaygene.showframe.do?projectid=defaultdb&db=kegg&recordid=62521629&loginid=BH11042&pathwayid=bta04310&gene=541127,NFATC3;505664,FRAT1;282041,ROCK2&pathwayname=Wnt+signaling+pathway+-+Bos+taurus+(cow)&pictureext=png) | 3 | 150 | 2.00% | 0.095 | 0.030 |
| [Antigen processing and presentation](http://sas.ebioservice.com/bioinfoplug_molnetpathwaygene.showframe.do?projectid=defaultdb&db=kegg&recordid=62521629&loginid=BH11042&pathwayid=bta04612&gene=510041,PSME1;281803,PDIA3&pathwayname=Antigen+processing+and+presentation+-+Bos+taurus+(cow)&pictureext=png) | 2 | 72 | 2.78% | 0.097 | 0.030 |
