# Supplementary material for: The Differential Expression of Immune Genes between Water Buffalo and Yellow Cattle Determines Species-Specific Susceptibility to Schistosoma japonicum Infection
Source: PLoS One. 2015 Jun 30;10(6):e0130344. doi: 10.1371/journal.pone.0130344 (PMC4488319; doi:10.1371/journal.pone.0130344)
Supplement: S10 Table — (DOC) [file pone.0130344.s010.doc]

**S10 Table. Primers in r**eal-time PCR validation for microarray results

| **Accession number** | **Gene symbol** | **Gene name** | **Primer(5‘-3’)** |
| --- | --- | --- | --- |
| **the DE genes both in yellow cattle and water buffalo post infection compared to pre infection** | | | |
| NM_001033617 | CTSC | cathepsin C | F:caaacctgcaccaatcactg R:gatcggggtctgagtgttg |
| AB098980 | ND1 | NADH dehydrogenase subunit 1 | F:acgacccgctacatcttcag R:tcgtagggctccgattagtg |
| NM_001098958 | CDKN1A | cyclin-dependent kinase inhibitor 1A | F:ctaacccccaactcagtggac R:ggtgtcacactgtccctcctg |
| NM_001076517 | LY6D | lymphocyte antigen 6 complex, locus D | F:ggtctctgcagaaggtctgg R:acgctgggctttgaatag |
| NM_001102498 | NKAPL | NFKB activating protein-like | F:gttaggaaggcaggggtaggc R:cgttggaatacccaagcac |
| NM_001038611 | ZPBP | zona pellucida binding protein | F:acgatgtcctgagtgctgtg R:atgcttttgctccaaacaccag |
| **The DE genes in water buffalo post- infection compared to pre infection** | | | |
| BC149477 | IL12RB1 | interleukin 12 receptor, beta 1 | F:ttctttactgccgagccac R:gatgtgaccttggccttgttgg |
| NM_001081520 | LY6G6E | lymphocyte antigen 6 complex, locus G6E | F:ctcagcgtgcagatcacattc R:ggtagcagggcttgacaaag |
| NM_176872 | THBS2 | thrombospondin 2 | F:gcttcgtccgctttgactac R: taggtgaggtccagggtgtc |
| **The DE genes in yellow cattle post-infection compared to pre infection** | | | |
| NM_001206292 | ZMYM6 | PREDICTED: Bos taurus zinc finger, MYM-type 6 | F:ctccatatgccttgggaaaatc R:atggccaggtgatactgagg |
| NM_001100304 | GPR52 | GPR52,G protein-coupled receptor 52 | F:atcaactggtcaccccatg R:gcagcaggggcataaag |
| NM_174006 | CCL2 | CCL2,chemokine (C-C motif) ligand 2 | F:cgcctgctgctatacattc R:gctcaaggctttggagtttg |
| NM_001192973 | AGMO | Bos taurus alkylglycerol monooxygenase (AGMO),TMEM195 | F:gagacaatccgtcctccaaatttac R:tcagttccaaaggaccaagg |
| XM_002694054 | TNFRSF8 | PREDICTED: Bos taurus Tumor necrosis factor receptor superfamily member 8-like | F:gttctacgccaggagctgag R:cttctccacgaggtctccag |
| **The common DE genes in yellow catte and water buffalo post infection compared to pre infection** | | | |
| NM_001077991 | RECQL | RecQ protein-like (DNA helicase Q1-like) | F:actgatcgactcctggatgg R:tgcgtgggcttctttactc |
| NM_001014956 | NFYA | NFYA,nuclear transcription factor Y, alpha | F:tgccaaacagtaccaccg R:gcttgatttggatcctgcatatg |
| NM_174452 | ROCK2 | Rho-associated, coiled-coil containing protein kinase 2 | F:tgaagcctgacaacatgctc R:gtaaccatcacccccttgtg |
| **The DE genes of water buffalo compared to yellow cattle at 7w post infection** | | | |
| NM_001080247 | JKAMP | JNK1/MAPK8-associated membrane protein | F:cagttggtggaggccttttatac R:caatgccaaaagcggtaaatc |
| NM_001034492 | C2 | Bos taurus complement component 2 | F:gtcagataccgctgctcctc R:cggaacgctggatttgtattttg |
| NM_175703 | EBD | Bos taurus defensin, beta 1 | F:ctccaccaccgactacagg R:aatcctgacccagcagacag |
| NM_174093 | IL1B | interleukin 1, beta | F:cagtgcctacgcacatgtctc R:agaggaggtggagagccttc |
| NM_173925 | IL8 | interleukin 8 | F:tcgatgccaatgcataaaaac R:cttttccttggggtttaggc |
| NM_174008 | CD14 | CD14 molecule | F:gcagcctggaacagtttctc R: tcctcaagcgtcagttccttg |
